# Delineating Metabolic Dysfunction-Associated Steatotic Liver Disease and Comorbid Risk With Clinical, Blood and Metabolomic Parameters—A UK Biobank Analysis

**DOI:** 10.1016/j.jceh.2026.103537

**Published:** 2026-04-07

**Authors:** Brian Ho, Yun Xu, Christopher E. Goldring, Royston Goodacre, Munir Pirmohamed

**Affiliations:** ∗ Wolfson Centre for Personalised Medicine, Institute of Systems, Molecular and Integrative Biology, University of Liverpool, Liverpool, UK; † Department of Pharmacology and Therapeutics, Institute of Systems, Molecular and Integrative Biology, University of Liverpool, Liverpool, UK; ‡ Centre for Metabolomics Research, Department of Biochemistry, Cell and Systems Biology, Institute of Systems, Molecular and Integrative Biology, University of Liverpool, Liverpool, UK

**Keywords:** NAFLD, MASLD, learning models, risk, comorbidity

## Abstract

**Background/objectives:**

Ascertaining risk of and prognostication tools for metabolic dysfunction-associated steatotic liver disease (MASLD) remain suboptimal. Using the UK Biobank, we investigated the role of multivariate supervised learning in predicting MASLD risk and the presence of disease subgroups in stratifying the risk of comorbid outcomes.

**Methods:**

Two ground truth definitions for MASLD were derived using magnetic resonance imaging–proton density fat fraction (MRI-PDFF) and electronic health records (EHRs). A training and validation set was created (60:40 split), using multiple imputation for missing data (mean missingness: 2.05%). Variable importance analyses of partial least squares-discriminant analysis (PLS-DA) and random forest (RF) were used to select important clinical, genetic, haematological, biochemical, and metabolomic features of MASLD, which were subsequently used to train models to classify disease risk. The presence of MASLD subgroups were explored by K-means clustering and the relationship with comorbid disease outcomes were investigated.

**Results:**

Three thousand seven hundred ninety-nine and 2552 individuals were available for analysis for the two ground truth definitions. A total of 49 features were selected to train our final models. Model performance based on area under receiver operating characteristics for PLS-DA, RF, support vector machine linear kernel, and logistic regression were all above ≥0.80 (MRI-PDFF definition) and ≥0.85 (MRI-PDFF and EHR definition) at predicting MASLD. Unsupervised clustering of the features in MASLD identified two risk subgroups with a ∼3-fold difference in ischaemic stroke, hypertension, and extrahepatic malignancy, a ∼2-fold difference in hyperlipidaemia and T2DM, and a >1.5-fold difference in ischaemic heart disease and all-cause mortality.

**Conclusions:**

Using multivariate supervised learning models, we have identified features of MASLD in UK Biobank participants that can be used to predict the risk of comorbid diseases and outcomes. This provides insights into MASLD pathophysiology and highlights the potential of learning models for case identification and prognostication. Further external validation is required to assess applicability of the predictive features and for model optimisation.

## Graphical abstract

**Figure:**
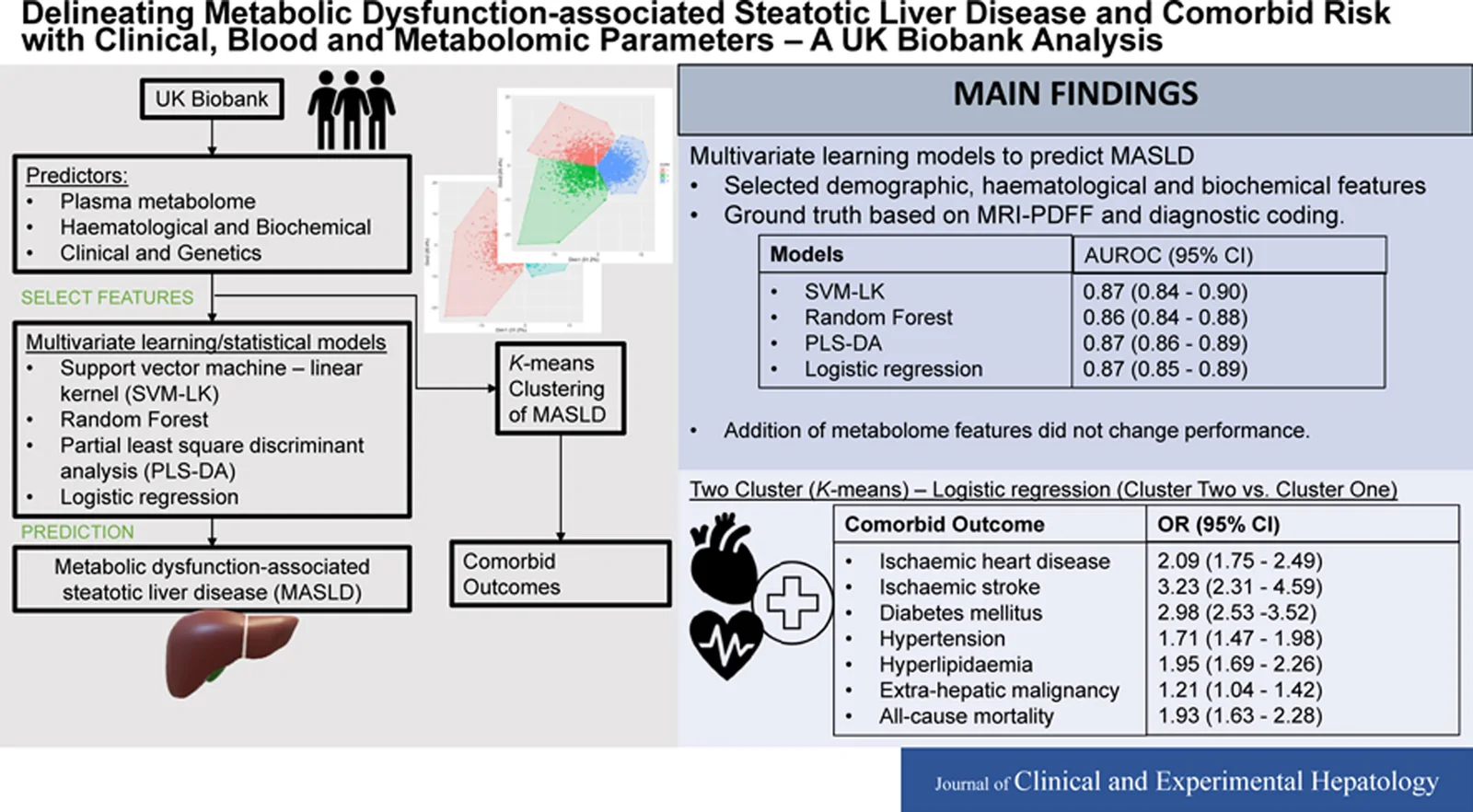

Nonalcoholic fatty liver disease (NAFLD), now termed metabolic dysfunction–associated steatotic liver disease (MASLD), is a growing healthcare burden affecting 30% of the global population.^1^ The nomenclature has recently changed to acknowledge the key pathogenic role of metabolic dysfunction, and the importance of a positive diagnosis, rather than of exclusion.^2^^,^^3^ Despite the intended benefits, this has created anxiety that previous research based on the old NAFLD definition may be rendered obsolete, but recent reports have reassuringly shown that the terminology is largely interchangeable.^4^

With metabolic dysfunction being paramount to the development and progression of MASLD, metabolome profiling from liver tissue, plasma, serum, and urine has generated valuable insights regarding the biological processes underpinning the disease.^5^ In one study, the levels of branch chain amino acids and aromatic amino acids in the plasma changed with disease severity.^6^^,^^7^ In another, the cumulative burden of genetic variants associated with steatohepatitis and fibrosis increased the level of metabolites related to mitochondrial redox states.^7^ The metabolome may thus augment our ability to interrogate the biological processes implicated in MASLD, such as genotype-phenotype effects.^8^^,^^9^ As such, genetics and metabolome data hold great potential in identifying the presence and stage of disease, which can be used as clinical tools for screening and prognostication.^6^^,^^10^

In this paper, we have explored the plasma metabolome along with clinical characteristics, genetics data and common haematological and biochemical assay results to predict the risk of MASLD. We defined MASLD using both magnetic resonance imaging–proton density fat fraction (PDFF) and diagnostic codes from electronic health records (EHRs), which are complementary for case finding. Multivariate supervised learning models (MVLM) to select the predictors, termed features, and train predictive algorithms in the UK Biobank (UKB) cohort, and subsequently internally assessed models’ performance. Using the selected features, we further explored the possible presence of MASLD patient subgroups and their risk of cardiovascular, metabolic, malignant, and mortality outcomes.

## MATERIALS AND METHODS

### UK Biobank Study

The UKB is a prospective cohort study with ∼500,000 individuals (40–69 years of age) from the United Kingdom, recruited between 2006 and 2010.^11^ The study gathered extensive phenotypic and genetic data from its participants, which included physical, behavioural, and clinical characteristics, assay results from biological samples (blood/saliva/urine), clinical bedside test results, and imaging data, with linked information to inpatient hospital and primary care records EHR.^12^ Ethics approval was obtained from the UK North-West Multi-Centre Ethics Committee (ref: 16/NW/0274). The following data analysis was conducted under the application ID 54764.

### Study Cohort and MASLD Definitions

Two ground truth MASLD definitions were used as previously published with some slight modifications.^13^ Briefly, the first definition used both ICD9/10 and primary care diagnostic coding from the EHR along with MRI-PDFF quantification of the liver provided by a more recent release of data field 24352 to determine the presence of MASLD.^14^ The second definition only used the MRI-PDFF measurement for the case definition. An MRI-PDFF cutoff of ≥5% was used. For both definitions, healthy individuals were defined as <5% MRI-PDFF, rather than the absence of a relevant diagnostic code to avoid inclusion of subclinical cases. The two definitions generated two sample populations referred to henceforth as MRI-EHR and MRI cohorts, respectively. Participants with an alcohol consumption of >21 (male) and >14 units (female) per week based on patient survey results, a recorded diagnostic code for another liver disease, previous liver transplantation, and a positive serum Hepatitis B/Hepatitis C virus serology, were excluded from the analysis. Although the study was conducted before the transition towards the MASLD definition, we have decided to update our terminology given the similarities between the two both diagnostically and prognostically.^2^

### Features of Interest

Clinical (*n* = 8), haematological, and biochemical (*n* = 65), and genetic (*n* = 7) features determined *a priori* were extracted from the UKB cohort [Table S1]. Plasma metabolome features were obtained: the data fields numbered 23400 to 23648 (249 measures), with 168 metabolite concentrations and 81 metabolite ratio derivatives extracted for the first 120,000 tranche of data release by the UKB. The metabolites measured consisted of amino acids, lipid, lipoproteins and their compositions or chemical properties. The detailed collection, processing, and derivation of the plasma metabolome have been detailed elsewhere.^15^

### Missing Data and Imputation

Multiple imputation with chained equations was used to impute features with missing values (*n* = 144, 38% of features) in individuals who had some or all available metabolome data [Supplementary Methods]. Three hundred thirteen and 10 features had <10% and 10–20% missing values, respectively. This amounted to 82,638 individuals for imputation after exclusion criteria were applied. MASLD status was not used in the imputation model to avoid introducing methodological bias. Highly correlated variables/features can reduce stability and precision of imputation estimates; therefore, for features with a pairwise correlation coefficient >0.7, only one was retained for use in the imputation matrix. For descriptive purposes, the correlation between metabolome measures is shown in Fig. S1. Five “complete” datasets were generated with predictive mean matching. As the design of multiple imputation allows within- and between-imputation variability to be accounted for, summary statistics were derived for individual imputed datasets and subsequently combined by applying Rubin’s rules in our learning model analyses. For our clustering analyses, the stacked “complete” datasets were used as part of the exploratory investigation into the presence of phenotypic subgroups of MASLD.

### Data Preprocessing

After imputation, individuals with determinable ground truth MASLD status were retained. Sixty percent of this dataset was selected by random and stacked together as the training set, with the rest used for validation for learning model assessment as the unstacked dataset. Training and validation sets were generated for each definition (MRI-EHR and MRI cohort), which overlapped in participants and provided a broad form of sensitivity analyses of learning models in training datasets with differing proportions of case and controls. Dummy variables were generated for categorical features. Range scaling was performed on the training set features, before applying it towards the validation set.

### Feature Selection

Partial least squares-discriminant analysis (PLS-DA) and random forest (RF) algorithms were trained using clinical characteristics and genetics, haematological and biochemical, and metabolomic features separately to identify individuals at risk of MASLD on the stacked imputed training set. Interrogation of scaled variable importance (value of 0–100) selected by the two model types (variable importance of projection in PLS-DA, Gini index in RF) allowed the identification of features that were of importance in classifying the MASLD risk. A feature was considered important if it was in the top 10 features ranked by their scaled importance and had a value of >50. Support vector machines with linear kernel (SVM-LK), RF, PLS-DA, and stepwise logistic regression were subsequently trained using the selected important features against both ground truth definitions. The value of metabolome features for MASLD was explored by assessing trained learning models with and without the metabolomic parameters.

### Training and Validation of Learning Models

The performance was optimised against receiver operating characteristics statistics on the training set. Hyperparameter optimisation was performed with grid search using 10-fold cross-validation. The area under the receiver operating curve (AUROC) was subsequently calculated against the separate imputed validation sets, with the 95% CIs generated by bootstrapping the five imputed test sets 2000 times. In a similar manner, positive and negative predictive values (PPVs and NPVs) were also calculated across prevalence ranges from 1% to 50% using case weighting. Final summary metrics were derived using Rubin’s rules. Hyperparameters for PLS-DA are the number of components; RF are the node size and number of features sampled at each split (mtry). A broad initial grid search was performed, before narrowing to a finer search to further optimise these hyperparameters. The initial hyperparameter search values were predetermined for each learning model type: RF node size and mtry (the square root of (*n* x 2)), PLS-DA components (0.9*n*), and SVM-LK C-value (5), whereby *n* denotes the number of selected features used to train the learning model. A general schema with explanation for feature selection and training and validation of learning models is shown in Supplementary Methods. We further derived AUROC measures for fatty liver index (FLI), derived as previously detailed, similarly on the test sets for comparison.^13^

### K-means Clustering

The presence of unique subgroups of individuals based on the selected predictive features was explored. Using the full imputed MRI-EHR cohort (combined training and validation set), individuals with MASLD were retained, to maximise sample size. All data were standardised (mean: 0 and standard deviation: 1) prior to clustering. The optimal number of clusters, *k*, was determined by inspection of elbow plot, silhouette measure, and gap statistics. We tested these methods for up to 20 clusters. In case of uncertainty between optimal *k*-values (i.e., cluster number) between different optimisation methods, all potential “optimal” *k*-clusters were considered.

### Cluster Outcome Analysis

Univariate logistic regression was used to investigate relationships between clusters and clinical outcomes of interest (ischaemic heart disease, ischaemic stroke, hepatic and extrahepatic malignancy, dyslipidaemia, hypertension, and all-cause mortality). The outcomes were extracted up to 31 December 2019, from the linked inpatient hospital and primary care records, based on ICD 9/10 and primary care codes as previously published.^13^ Individuals with prevalent disease (i.e. same as the outcome) was excluded from the analysis. The difference in cluster risk towards outcomes was considered significant if it achieved a *P*-value <0.05, with Bonferroni correction (0.05/7 tests).

### Statistical Analysis

Statistical analysis was mainly performed using R v.4.1.2. For training of models with demographic and genetics data, and haematology, biochemistry, and metabolome features, R v.3.6.0 was utilised on a server system (Red Hat Enterprise Linux OS) hosted by the University of Liverpool. R packages used for analyses and access to R scripts used are detailed in Supplementary Methods**.**

## RESULTS

A total of 82,638 individuals had all or partial measurements of their metabolome from the UKB and underwent multiple imputation for missing values. Within this sample population, 3799 and 2552 individuals had both metabolome and clinical information allowing ground truth determination of the MASLD disease status using the MRI-EHR and MRI definitions, respectively. The characteristics of these two cohorts are summarised in Tables 1 and S2 and generally followed the previously observed pattern of distribution expected for MASLD. Splitting the cohort into training and validation sets (60:40) resulted in 2280 and 1532 individuals in the MRI-EHR and MRI cohort for model training, respectively. Details of hyperparameters used for all trained algorithms detailed hereafter are shown in Table S3.

**Table 1:** Characteristics of MRI-PDFF and Electronic Diagnostic Records-defined (MRI-EHR) Cohort.

Characteristic	Healthy	*n*	MASLD	*n*
Clinical				
Age (Years)	55 (48–61)	1929	57 (50–62)	1870
Sex (male)	768 (39.8%)	1929	1624 (42.8%)	1870
Smoking				
Ex-smoker	499 (25.8%)		1121 (29.5%)	
Current	81 (4.2%)	1928	183 (9.8%)	1868
BMI	25.08 (23.05–27.69)	1927	30.09 (27.12–34.15)	1860
Waist-hip ratio	0.50 (0.46–0.54)	1928	0.58 (0.54–0.64)	1862
Systolic blood pressure (mmHg)	127 (117–139)	1929	133 (123–145)	1864
Diastolic blood pressure (mmHg)	77 (71–84)	1929	82 (75–88)	1865
Alcohol (units/week)	8.20 (4.80–11.85)	1442	7.75 (3.06–11.88)	1130
Townsend deprivation index	−2.91 (−4.03–1.01)	1924	−1.74 (−3.46–1.31)	1867
Serum				
Direct bilirubin (μmol/L)	1.58 (1.28–2.07)	1570	1.61 (1.29–2.09)	1480
AST (U/L)	23.6 (20.35–27.10)	1847	26.90 (22.30–33.50)	1763
ALT (U/L)	17.94 (14.23–23.48)	1854	27.24 (19.87–39.44)	1768
ALP (U/L)	76.8 (63.92–91.35)	1854	86.35 (71.90–104.43)	1768
GGT (U/L)	21 (15.7–30.1)	1853	37.10 (25.10–59.45)	1767
Albumin (g/dL)	45.28 (43.52–46.93)	1692	44.99 (43.21–46.88)	1633
Apolipoprotein A (g/L)	1.52 (1.35–1.7)	1685	1.39 (1.26–1.55)	1625
Apolipoprotein B (g/L)	1.01 (0.85–1.17)	1846	1.03 (0.87–1.21)	1756
HBA1C (mmol/mol)	34.40 (32.20–36.70)	1839	37.10 (33.82–40.60)	1778
Platelets (10^9^ cells/L)	246.30 (213.30–282.30)	1879	254 (214.50–295.90)	1817
HDL-C (mmol/L)	1.44 (1.23–1.70)	1694	1.20 (1.03–1.41)	1631
LDL-C (mmol/L)	3.53 (2.99–4.12)	1852	3.48 (2.87–4.17)	1764
Cholesterol (mmol/L)	5.64 (4.93–6.44)	1853	5.48 (4.68–6.31)	1768
Triglyceride (mmol/L)	1.3 (0.94–1.89)	1851	1.94 (1.41–2.74)	1768
Lipoprotein-A (nmol/L)	21.8 (10.00–60.02)	1495	19.23 (8.72–54.76)	1366
IGF1 (nmol/L)	22.29 (18.63–25.43)	1846	20.55 (16.33–24.32)	1758
Urate ((μmol/L)	275.3 (229.12–328.45)	1846	331.40 (281.40–382.80)	1769

Table shows the clinical, serum haematological, and biochemical characteristics of the cohort prior to imputation where the MASLD and healthy individuals were defined by MRI-PDFF and electronic health records (n = 3799). Continuous variables are shown as median and interquartile range, whilst categorical data are shown in frequency (%). ALT – alanine aminotransferase, ALP – alkaline phosphatase, AST – aspartate aminotransferase, BMI – body mass index, EHR – electronic health record, GGT – gamma-glutamyl transpeptidase, HBA1c – glycated haemoglobin, HDL-C – high density lipoprotein cholesterol, IGF-1 – Insulin growth factor-1, LDL-C – low density lipoprotein cholesterol, MASLD – metabolic dysfunction-associated steatotic liver disease, MRI-PDFF – magnetic resonance imaging–proton density fat fraction.

### Clinical and Genotype Features of MASLD

**Figure 1:**
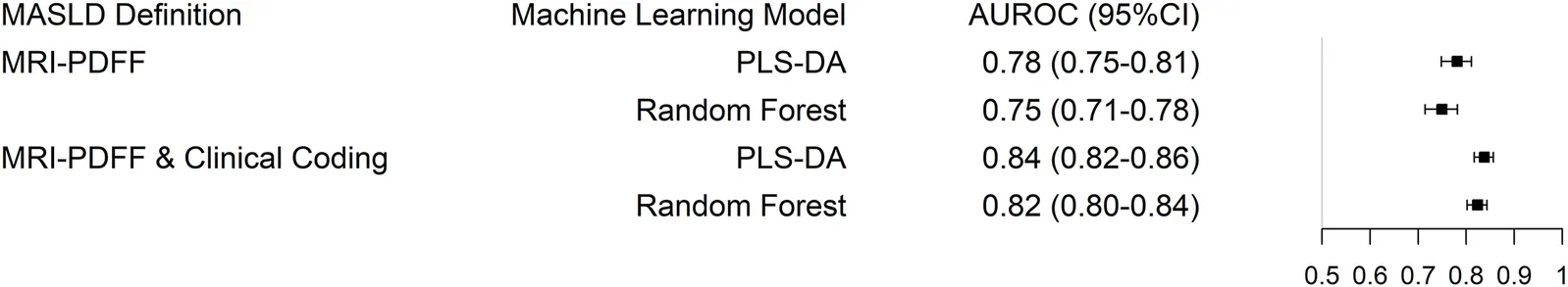
**– Performance of multivariate supervised learning models trained with demographic and genetic features.** Partial least squares-discriminant analysis (PLS-DA) and random forest algorithms were trained against MASLD cases defined by MRI-PDFF, electronic health records, and MRI-PDFF. AUROC (area under curve of receiver operating characteristics) and 95%CI are shown by the forest plot. CI – confidence interval, MASLD – metabolic dysfunction-associated steatotic liver disease, MRI-PDFF – magnetic resonance imaging–proton density fat fraction.

We trained PLS-DA and RF models for both MASLD definitions using clinical characteristics and genotype features. Using our feature selection criteria, age, waist-hip ratio, body mass index (BMI), sex, smoking status, systolic blood pressure, and Townsend index were selected as important clinical predictors of MASLD risk. In contrast, none of the genetic features were selected [Fig. S2–3]. In the MRI-EHR validation cohort, this corresponded to AUROCs of 0.84 (95% CI: 0.82–0.86) and 0.82 (95% CI: 0.80–0.84), from the PLS-DA and RF algorithms, respectively. A lower performance for each of these respective algorithms was seen in the same trained models for the MRI cohort [AUROC 0.78 (95%CI: 0.75–0.81) and 0.75 (95%CI: 0.71–0.78)] [Figure 1 and S4-S7]**.**

### Haematological and Biochemical Features of MASLD

**Figure 2:**
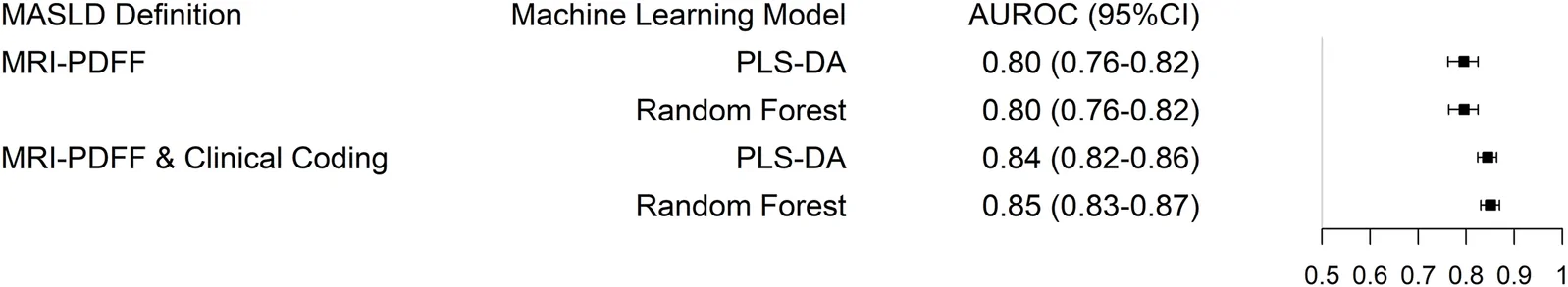
**Performance of multivariate supervised learning models trained with serum haematological and biochemistry features.** Partial least squares-discriminant analysis (PLS-DA) and random forest algorithms were trained against MASLD cases defined by MRI-PDFF and electronic health records and MRI-PDFF. AUROC and 95%CI are shown by the forest plot. AUROC – area under receiver operating characteristics curve, CI – confidence interval, MRI-PDFF – magnetic resonance imaging–proton density fat fraction, MASLD – metabolic dysfunction-associated steatotic liver disease.

A similar approach was applied for the haematological and biochemical blood results. PLS-DA and RF gave an AUROC of 0.84 (95%CI: 0.82–0.86) and 0.85 (95%CI of 0.83–0.87), respectively, using the MRI-EHR definition. Similarly, in the MRI validation cohort, the AUROC was again comparatively lower in the respective algorithms [0.80 (95%CI: 0.76–0.82) and 0.80 (95%CI: 0.76–0.82)] [Figure 2 and S8-11]. The important haematological and biochemical features which classified MASLD risk from both definitions and algorithms were gamma-GT, alanine aminotransferase, aspartate aminotransferase, high density lipoprotein cholesterol, sex hormone-binding globulin, urate, apolipoprotein A, C-reactive protein, urinary sodium levels, glycated haemoglobin high light-scattered reticulocyte, high light reticulocyte count, immature reticulocyte fraction, and reticulocyte count [Fig. S12–13].

### Metabolome Features of MASLD

**Figure 3:**
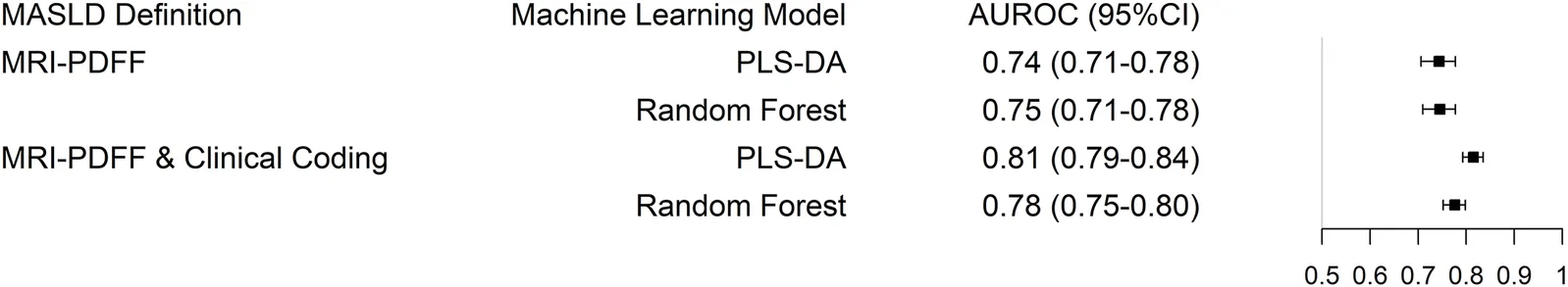
**- Performance of multivariate supervised learning models trained with metabolome features.** Partial least squares-discriminant analysis (PLS-DA) and random forest algorithms were trained against MASLD cases defined by MRI-PDFF and electronic health records and MRI-PDFF. AUROC and 95%CI are shown by the forest plot, derived by applying Rubin’s rules. AUROC – area under receiver operating characteristics curve, CI – confidence interval, MRI-PDFF – magnetic resonance imaging–proton density fat fraction, MASLD – metabolic dysfunction-associated steatotic liver disease.

Finally, the 249 metabolome measures available in the UKB were used to train PLS-DA and RF models for the MRI-EHR and MRI cohort, respectively. The validation set performance from PLS-DA and RF gave AUROCs of 0.81 (95%CI: 0.89–0.84) and 0.78 (95%CI: 0.75–0.80), respectively, in the MRI-EHR validation cohort. With the MRI cohort, the AUROCs for the PLS-DA and RF algorithms were 0.74 (95%CI: 0.71–0.78) and 0.75 (95%CI: 0.71–0.78), respectively [Figure 3 and S14-17]. The top 10 features selected were mainly lipid levels including triglycerides, cholesterol, and lipoproteins, along with their compositional measures. Two amino acids (glycoprotein acetyls and branch chained amino acids concentrations) also met our feature selection criteria [Fig. S18–19].

### Classifying MASLD with Combined Features

**Figure 4:**
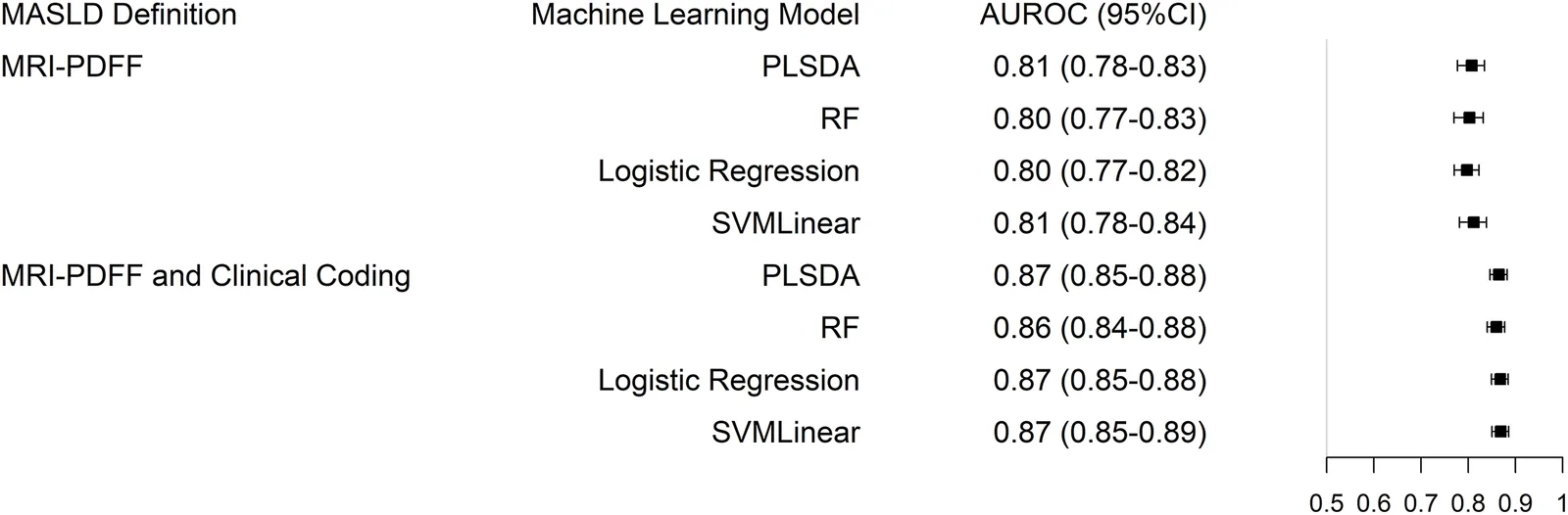
**- Performance of multivariate supervised learning models trained with demographics and serum haematological and biochemistry features.** Partial least squares-discriminant analysis (PLS-DA), random forest (RF), support vector machine (SVM) linear kernel and logistic regression algorithms were trained against MASLD cases defined by MRI-PDFF and electronic health records and MRI-PDFF. AUROC and 95%CI are shown by the forest plot. AUROC – area under receiver operating characteristics curve, CI – confidence interval, MRI-PDFF – magnetic resonance imaging–proton density fat fraction, MASLD – metabolic dysfunction-associated steatotic liver disease.

The final list of selected features is shown in Table 2. We first trained four multivariate supervised learning models using the same cohort (including same training-test set splits) with selected clinical, haematological, and biochemical features. Better performances were again observed against the validation MRI-EHR cohort compared to the MRI cohort for the trained algorithms [Figure 4 and S20-27]. All models performed similarly with overlapping 95% confidence intervals (CIs) of AUROC. F1, PPVs, and NPVs across prevalence ranges were calculated [Table S4 and S6]. With a MASLD prevalence of 30%, the mean PPV and NPV were 88–90% and 49–53% for the MRI cohort and 90% and 61–63%, respectively.

**Table 2:** Final List of Selected Features for Multivariate Supervised Learning Algorithm Predictors Between Both NAFLD Ground Truth Definitions.

Feature type	Selected features
Clinical	BMI
	Waist-hip ratio
	Townsend index
	Age
	Sex
	Systolic blood pressure
Haematological and biochemical	Gamma-glutamyl transpeptidase
	Alanine aminotransferase
	Triglyceride
	High light-scattered reticulocyte
	High light reticulocyte
	High-density lipoprotein cholesterol
	Sex hormone-binding globulin
	Urate
	Apolipoprotein A
	C-reactive protein
	Urinary sodium
	Glycated haemoglobin
	Reticulocyte count
Generalised metabolome characteristics	Cholesterol in medium VLDL
	Cholesterol in small LDL
	Cholesterol in very large VLDL
	Cholesterol to total lipids in large HDL percentage
	Cholesterol to total lipids in large LDL percentage
	Cholesterol to total lipids in very small VLDL percentage
	Cholesteryl esters in very large HDL
	Cholesteryl esters to total lipids in large HDL percentage
	Cholesteryl esters to total lipids in large LDL percentage
	Concentration of large HDL particles
	Glycoprotein acetyls
	Free cholesterol to total lipids in large HDL percentage
	Linoleic acid to total fatty acids percentage
	Omega-6 fatty acids to total fatty acids percentage
	Phospholipids in LDL
	Phospholipids in very large HDL
	Phospholipids to total lipids in large HDL percentage
	Polyunsaturated fatty acids to monounsaturated fatty acids ratio
	Polyunsaturated fatty acids to total fatty acids percentage
	Total cholesterol minus HDL-C
	Total concentration of branched-chain amino acids (Leucine + isoleucine + Valine)
	Total lipids in large HDL
	Total lipids in LDL
	Total lipids in medium LDL
	Total lipids in small LDL
	Total lipids in small HDL
	Total lipids in very small VLDL
	Total lipids in very large HDL
	Total lipids in very large VLDL
	Triglycerides in large LDL
	Triglycerides in LDL
	Triglycerides to total lipids in large LDL percentage
	Triglycerides to total lipids in very small VLDL percentage
	VLDL cholesterol

HDL – high density lipoprotein, LDL – low density lipoprotein, NAFLD – nonalcoholic fatty liver disease, VLDL – very-low-density lipoprotein.

**Figure 5:**
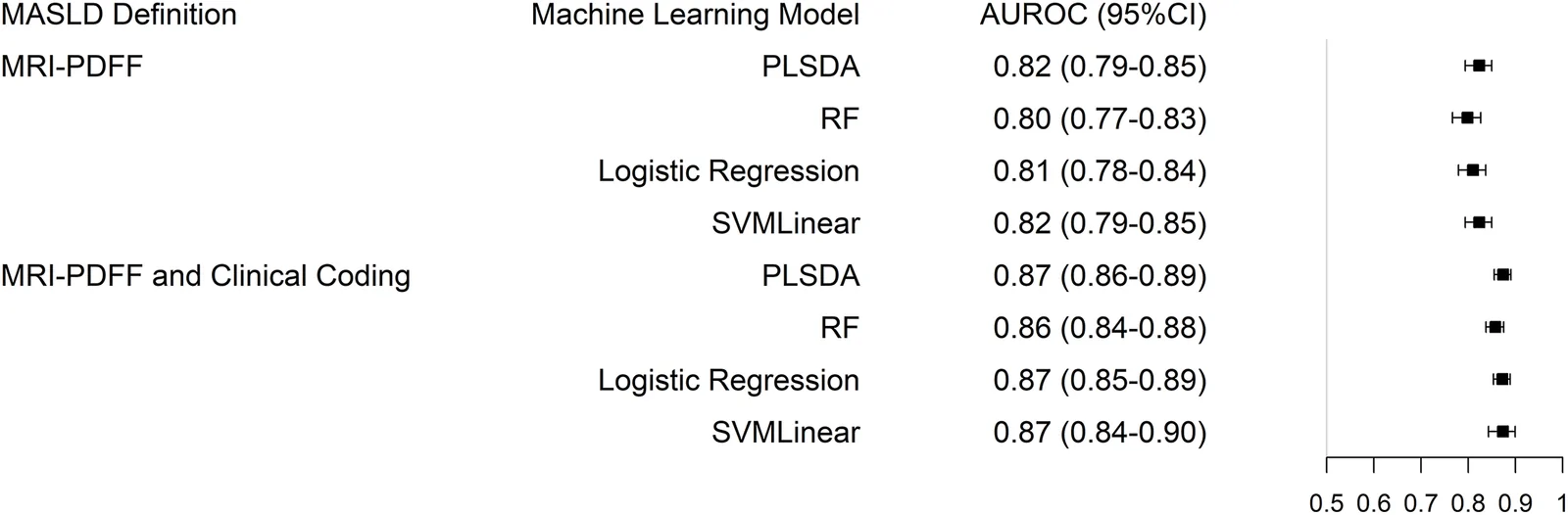
**– Performance of multivariate supervised learning models trained with demographics, serum haematological and biochemistry, and plasma metabolome features.** Partial least squares-discriminant analysis (PLS-DA), random forest (RF), support vector machine (SVM) linear kernel, and logistic regression algorithms were trained against MASLD cases defined by MRI-PDFF and electronic health records and MRI-PDFF. AUROC and 95%CI are shown by the forest plot. CI – confidence interval, MRI-PDFF – magnetic resonance imaging–proton density fat fraction, MASLD – metabolic dysfunction-associated steatotic liver disease.

Further multivariate supervised learning models were trained with the addition of selected generalised metabolome features [Figure 5 and S28-35]. We refer to these as generalised metabolome characteristics as these are not specific metabolite concentrations but combinations of features [Table 2]. Once again, the models had similar performance based on their ROC. Addition of selected metabolome features did not improve the model using clinical characteristics, haematological, and biochemical features. For comparison, FLI the performance across both definitions were (AUROC (95% CI): MRI-EHR cohort 0.84 (0.82–0.86) and MRI-PDFF cohort 0.78 (0.75–0.81). The frequency of MASLD and healthy individuals by predicted probability of disease is plotted in the histograms for each learning model for descriptive purposes using one of the imputed validation sets [Fig. S36–37]. Given an estimated MASLD prevalence of 30%, the mean PPV and NPV was 81–91% and 50–62% for MRI cohort and 88–90% and 57–69%, respectively. F1, PPV, and NPV were also calculated across reference ranges [Table S5 and S7]. Regressors selected by stepwise logistic regression are further shown in [Table S8].

### Features May Delineate Risk of MASLD-related comorbidities

**Figure 6:**
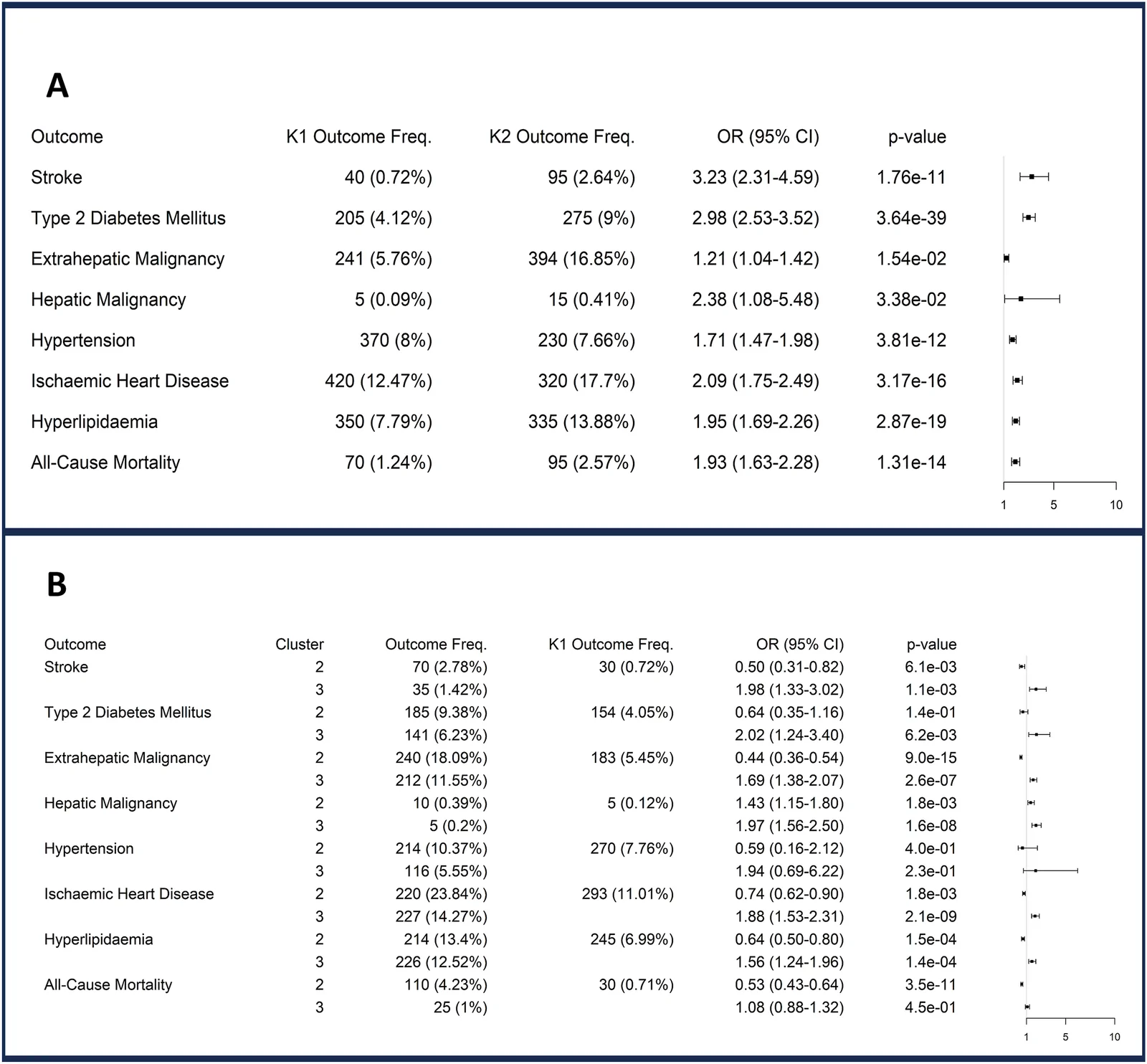
**Risk of comorbid outcomes by *k*-means clusters in MASLD**. The forest plot shows logistic regression analysis for clusters generated from K-means unsupervised clustering of individuals who develop MASLD. (A) Two and (B) three disease clusters with associated risk of disease outcomes are shown as odds ratio with 95%CI, represented by forest plot’s dot and whiskers. The reference cluster is cluster one (K1). The proportion of individuals in each cluster that develop event of interest is also shown. CI – confidence interval, MASLD – metabolic dysfunction-associated steatotic liver disease.

Selected features that determine the risk of MASLD may also differentiate potential disease subgroups and this was explored using *k*-means clustering in individuals with MASLD from the MRI-EHR cohort. The elbow, silhouette, and gap statistics suggested two or three distinct clusters of MASLD based on these features. Using logistic regression analysis, we therefore explored the risk of incident comorbid diseases by clusters. With two clusters, it was possible to differentiate the risk of all comorbid conditions, except for hepatic malignancy [Figure 6]. With three clusters, the odds of extrahepatic malignancy and ischaemic heart disease differed completely between clusters, with no overlapping CIs in odds ratios. The risk of other outcomes was partially differentiated except for hypertension where incident risk did not differ among the three clusters. The median follow-up period ranged from 930 days for ischaemic disease to 3352 days for all-cause mortality [Table S9].

## DISCUSSION

In this study, we leveraged MVLMs to identify complex data patterns to classify the risk of MASLD and its comorbidities. We first utilised two model types, RF and PLS-DA, to interrogate variable importance of clinical, genetics, haematological and biochemical, and metabolomics features separately, to then select a smaller “important” set of features to train our final models. Our final logistic regression model and more complex models (PLS-DA, RF, and SVM-LK) showed superiority in predicting the risk of developing MASLD compared to FLI. FLI was chosen for comparison as we showed previously that it identified MASLD better than hepatic steatosis index and lipid accumulation product.^13^ By the nature of UKB’s study design, it suggests measures collected at the inception of the study predict the risk of pre-existent or future MASLD, implying that predisposition is largely captured by clinical parameters in middle-early late age. Furthermore, unsupervised clustering of MASLD individuals using the selected features, showed the potential of distinct disease subgroups with different risks of developing comorbidities and all-cause mortality.

During variable importance assessment, age, sex, waist-hip ratio, BMI, Townsend index, and systolic blood pressure were considered as important MASLD determinants, replicating the known epidemiological risk factors of the disease.^16^, ^17^, ^18^ In contrast, genetic variants known to be associated with MASLD were of low importance, consistent with the small effect sizes of the variants demonstrated in GWASs and were not selected in our final models.^19^ Furthermore, serum biochemical features that reflect the risk of developing MASLD, including markers of inflammation (C-reactive protein [CRP]), insulin resistance (glycated haemoglobin - HBA1c), liver injury (alanine aminotransferase and gamma-glutamyl transpeptidase, and lipid homoeostasis (apolipoprotein A), reflect the importance of metabolic syndrome in MASLD pathogenesis. Other selected features included sex hormone-binding globulin and urine sodium. This may stem from the known contribution of androgens in insulin resistance states, and a recent study has suggested a sex-dependent association of testosterone levels with MASLD.^20^ Two previous studies have also suggested that the urinary sodium level increases the risk of MASLD reflecting dietary sodium intake.^21^^,^^22^

When exploring pertinent metabolomic features, fatty acid and lipid species fractions were predominantly selected but this may result from the large proportion of lipid, cholesterol, and fatty acid measurements conducted by UKB. However, their close relationship with hepatic accumulation of lipids and cholesterol make them plausible features of importance.^6^^,^^10^^,^^23^ Beyond lipid measures, branched-chain amino acids and glycoprotein acetyl levels were also selected as important predictors. In humans, branched-chain amino acids are not synthesised in the body and hence daily requirements are solely supplied through diet.^24^ Previous studies have shown that levels are elevated in individuals who are obese^25^ and/or are insulin resistant.^26^^,^^27^ Moreover, mouse models have suggested that branched-chain amino acids have diverse systemic effects and contribute to systemic adiposity and insulin resistance states.^28^^,^^29^ In MASLD, plasma metabolome profiling showed that individuals with histological steatosis and nonalcoholic steatohepatitis also had increased levels of branched-chain amino acids compared to controls.^6^ By contrast, glycoprotein acetyls are markers of systemic inflammation and cardiovascular risk. A previous study using nuclear magnetic resonance quantification of serum metabolome showed that increased glycoprotein acetyl levels correlated with MASLD in an adolescent, young, and middle-aged cohort.^30^

Utilising important clinical characteristics, serum haematological and biochemical parameters, with or without metabolomic predictive features, logistic regression, SVM-LK and RF, and PLS-DA algorithms performed equally well. However, addition of metabolome features (AUROC: 0.86–0.87) did not improve the overall model performance compared to without their inclusion (AUROC: 0.86–0.87), with overlapping CIs, suggesting intersection of risk information captured by metabolome and other included features. For instance, the inclusion of biochemically determined triglyceride level likely captures the same associative risk towards MASLD originating from lipid metabolite measures. This likely stems from our features selection strategy, whereby we interrogated demographics, haematology and biochemistry, and metabolome features separately. Notably, when training SVM-LK models against the MRI-EHR cohort, we did encounter instances whereby classification probabilities could not be calculated. This is likely explained by the failure of Platt scaling, which estimates the output of SVM-LK onto a sigmoid probability curve and is a known problem with this learning algorithm.^31^^,^^32^

Overall, our trained models seemed to perform better with the MRI-EHR cohort than the MRI cohort. The former had a more balanced number of MASLD and healthy individuals, representing a more optimal dataset for training models, potentially explaining this difference. For practical applications, logistic regression models will be of greater appeal as they require less computational power to train when compared to other learning models.^33^^,^^34^ RF does, however, have the advantage of scaling and generally has better accuracy in different populations.^34^

As an overarching term, MASLD likely encompasses a heterogenous disease population with patients harbouring variable combinations of genetic and environmental risk factors.^35^ This has resulted in crude stratification of MASLD patients, such as lean MASLD, where the progression risk to advanced liver disease can differ.^37^, ^38^, ^39^ To explore this, we investigated the presence of MASLD subgroups that may implicate different risk of comorbid outcomes, based on the selected features used to train our final models. Applying *K*-means clustering indicated that MASLD may consist of two to three distinct disease subgroups. Using two clusters showed better risk differentiation than three clusters for cardiovascular, metabolic, malignant, and mortality outcomes. To our knowledge, one previous study has shown that MASLD can be divided into three subgroups by serum metabolome alone with differing incident risk of cardiovascular disease based on the Framingham risk score.^36^ Due to differences in variables used for clustering, experimental, and statistical methodology, it is difficult to directly compare results. Furthermore, the unsupervised nature of K-means, as opposed to supervised methods, the clusters are not optimised towards discerning our investigated disease outcomes. As such, prognostically distinct disease subgroups warrant further investigation.^39^ Lastly, collinearity of variables may create clustering bias and dimensionality reduction could reduce this prior to clustering. However, we omitted this for reasons of interpretability and the exploratory nature of our cluster analysis.

Our study has some general limitations. Firstly, the UKB study design limited our analysis to a cross-sectional design. However, our algorithms show that the risk of developing MASLD can be discerned with clinically gathered information at middle or early late age. Secondly, without an external validation cohort, the results serve as a starting point for further scrutiny. The difficulty lies in finding a separate patient cohort that measures the range of parameters in a similar manner available in the UKB and thus limited us to perform only internal validation. Thirdly, there is room to refine learning models presented here iteratively, to allow optimised feature selection such as recursive feature elimination, hyperparameter optimisation, and model calibration. The inclusion of all features during model derivation in this scenario will increase the required computational power.

In summary, we have shown that clinical characteristics, along with blood haematological, biochemical, and metabolomic parameters involved in the pathogenesis of MASLD may be useful in predicting the risk of its development. The addition of metabolome features did not improve the models possibly related to overlapping information stemming from our method of feature selection. Logistic regression, PLS-DA, SVM-LK, and RF models, outperformed FLI, with logistic regression being the more attractive practical option due to its relatively lower computational requirements. Importantly, the selected features also identified MASLD subgroups with a differential risk of developing comorbid diseases.

## Data availability

The data used for analysis were obtained from the UK Biobank and the processes of obtaining this are stated in http://www.ukbiobank.ac.uk/using-the-resource/. This research has been conducted under application number 54764.

## Disclosures

M.P. has developed a HLA genotyping panel with MC Diagnostics but does not benefit financially from this. He is part of the IMI Consortium ARDAT (www.ardat.org). Other investigators have no conflicts of interest to declare.

## Credit authorship contribution statement

Funding: MP, Manuscript concept: BH, CEG, YX, RG, and MP. Manuscript design and writing: BH. Statistical analysis: BH and YX. Revision, editing and acceptance of final version: all authors.

## Funding

BH was an MRC Clinical Training Fellow based at the University of Liverpool supported by the North West England Medical Research Council Fellowship Scheme in Clinical Pharmacology and Therapeutics, which is funded by the Medical Research Council (Award Ref. MR/N025989/1), Roche Pharma, Eli Lilly and Company Limited, UCB Pharma, Novartis, the University of Liverpool, and the University of Manchester. MP is principal investigator for this award. BH is also supported by a National Institute for Health and Care Research (NIHR) Academic Clinical Lectureship (CL-2025-07-501) in partnership with the NIHR Imperial Biomedical Research Centre [NIHR203323]. The funders had no role in the study design, data collection, analysis and interpretation, or writing of this manuscript.

## Declaration of competing interest

The authors declare that they have no known competing financial interests or personal relationships that could have appeared to influence the work reported in this paper.
