# Supplementary material for: Delineating Metabolic Dysfunction-Associated Steatotic Liver Disease and Comorbid Risk With Clinical, Blood and Metabolomic Parameters—A UK Biobank Analysis
Source: J Clin Exp Hepatol. 2026 Apr 7;16(4):103537. doi: 10.1016/j.jceh.2026.103537 (PMC13129434; doi:10.1016/j.jceh.2026.103537)
Supplement: Supplementary file 1 [file mmc1.docx]

**Title – Delineating Metabolic Dysfunction-associated Steatotic Liver Disease Risk and Subgroups Using Machine Learning – A UK Biobank Analysis**

**Authors:** Brian Ho^1,2^, Yun Xu^3^, Christopher E Goldring^2^, Royston Goodacre^3^, Munir Pirmohamed^1,2^

**Affiliations:**
1 - Wolfson Centre for Personalised Medicine, Institute of Systems, Molecular and Integrative Biology, University of Liverpool, Liverpool, UK
2 – Department of Pharmacology and Therapeutics, Institute of Systems, Molecular and Integrative Biology, University of Liverpool, Liverpool, UK.
3 - Centre for Metabolomics Research, Department of Biochemistry, Cell and Systems Biology, Institute of Systems, Molecular and Integrative Biology, University of Liverpool, Liverpool, UK

**Correspondence:
Brian Ho**
The University of Liverpool
The Wolfson Centre for Personalised Medicine
Block A: Waterhouse Buildings
1-5 Brownlow Street
Liverpool L69 3GL
Email: [b.ho@liverpool.ac.uk](mailto:b.ho@liverpool.ac.uk)

**Supplementary Materials**

| **Supplementary Methods** | **3-5** |
| --- | --- |
| **Table S1 – Demographic, genetic and biochemical data fields extracted from the UK Biobank.** | **6** |
| **Table S2 – Characteristics of MRI-PDFF defined cohort.** | **7** |
| **Table S3 - Hyperparameter selection through grid search of trained machine learning models.** | **8** |
| **Table S4 – Positive and Negative Predictive Value Across Prevalence Ranges of MASLD (DHB models)** | **9** |
| **Table S5 - Positive and Negative Predictive Value Across Prevalence Ranges of MASLD (All Feature Models)** | **10** |
| **Table S6 - F1 Scores of Models Across Prevalence Ranges of MASLD (DHB Feature Models)** | **11** |
| **Table S7 – F1 Scores of Models Across Prevalence Ranges of MASLD (All Feature Models)** | **12** |
| **Table S8 – Predictors selected by stepwise regression models** | **13** |
| **Table S9 - Median Follow-up period of all disease outcomes of interest** | **15** |
| **Figure S1 – Correlation Heatmap of Plasma Metabolome** | **16** |
| **Figure S2 - Variable Importance Plot (Scaled) for Partial Least Square Regression algorithm using demographic and genetic features.** | **17** |
| **Figure S3 - Variable Importance Plot (Scaled) for Random Forest algorithm using demographic and genetic features.** | **18** |
| **Figure S4-7 - Receiver operating characteristics curve of machine learning algorithms with demographics and genetics features to classify MASLD risk (defined by both ground truth definitions) of individual imputed validation sets.** | **19-22** |
| **Figure S8-11 – Receiver operating characteristics curve of machine learning algorithms with haematological and biochemical features to classify MASLD risk (defined by both ground truth definitions) of individual imputed validation sets.** | **23-26** |
| **Figure S12 - Variable Importance Plot (Scaled) for partial least square algorithms using haematological and biochemical features** | **27** |
| **Figure S13 - Variable Importance Plot (Scaled) for Random Forest algorithm using haematological and biochemical features.** | **28** |
| **Figure S14-17 – Receiver operating characteristics curve of machine learning algorithms with metabolome features to classify MASLD risk (defined by both ground truth definitions) of individual imputed validation sets.** | **29-32** |
| **Figure S18 - Variable Importance Plot (Scaled) for Partial Least Square algorithm using metabolomic features.** | **33** |
| **Figure S19 - Variable Importance Plot (Scaled) for Random Forest algorithm using metabolomic features.** | **34** |
| **Figure S20-27 – Receiver operating characteristics curve of machine learning algorithms with selected demographic, haematology and biochemistry features to classify MASLD risk (defined by both ground truth definitions) of individual imputed validation sets.** | **35-42** |
| **Figure S28-35 – Receiver operating characteristics curve of machine learning algorithms with selected demographic, haematology and biochemistry, and metabolome features to classify MASLD risk (defined by both ground truth definitions) of individual imputed validation sets.** | **43-50** |
| **Figure S36 – Classification probabilities of machine learning models trained with selected demographic and haematology and biochemistry features.** | **51** |
| **Figure S37 – Classification probabilities of machine learning models trained with selected demographic and haematology and biochemistry, and metabolome features.** | **52** |

**Supplementary Methods**

**MASLD Cohorts and Multiple Imputation**

As missing data is present in the UK Biobank, we utilised multiple imputation to impute missing values, with the assumption that missing mechanism is missing at random or missing completely at random. Multiple imputation has widely been used to reduce bias in results from missing data. The method briefly uses variables available as a model to impute missing values but imputes multiple “complete” datasets to counter imputation model variances (i.e. variation/inaccuracies) by allowing between and within model variances to be considered during analysis. We did not incorporate MASLD in the imputation model as we aim to train models to predict MASLD risk. Furthermore, incorporating the extended UK Biobank cohort with metabolome data available from the first data release, for imputation, allowed an increase in the imputation sample size. Predictive mean matching was the imputation method used and 5 datasets were imputed.

Within the UK Biobank, MASLD was defined by the MRI-PDFF criteria detailed in the methods section of the main article (MRI-PDFF >5%) with or without the use of electronic health records (e.g. a diagnostic code in the inpatient hospital record or GP records of MASLD). MRI-PDFF levels below the cutoff was used to define healthy individuals. This gave two datasets (MRI and MRI-EHR) for analysis. The former is inclusive of the latter and serves as the ground truth definition.

Analysis was done on the final cohorts with individuals that have ground truth definitions as with the schema directly below.

**Feature selection, training and validation of learning models.**

Models were trained with MRI and MRI-EHR cohort, of which each has now five imputed datasets. The datasets were split into training and validation set (60:40) and the five training sets were stacked for training partial least square discriminant analysis and random forest models. These two models were selected as they allow examination of whether predictive variables, also known as features, are important predictors of MASLD. Briefly, in random forest, the gini index was used which looks at how features change classification accuracies. Similarly, variable importance of projection for partial least square regression discriminant analysis assesses feature importance based on underlying partial least square coefficients. Further details are in the manuals for the R ranger package [<https://cran.r-project.org/web/packages/ranger/ranger.pdf>] and caret package [<https://cran.r-project.org/web/packages/caret/caret.pdf>].

After selection of features based on criteria set in our main methods section, the further models were trained with selected features with or without metabolomic features. As using the stacked dataset may increase over fitting, assessment of model performance was done on each imputed dataset separately assess true performance of the models, with the summary statistics combined using Rubin’s Rules, which accounts for intra- and inter dataset variances from the imputation model.

A general schema is shown directly below.

**Packages used for data analyses**

- dplyr (table manipulation)
- corrplot and ggcorrplot (correlation analysis)
- mice (multiple imputation)
- caret (multivariate supervised learning models)
- cluster and factoExtra (factor and cluster analysis)
- pROC (receiver operating characteristic analysis).
- psfmi (pool AUC values from imputed datasets) - ran R v.4.0.0 due to package compatibility.

**Table S1 – Demographic, genetic and biochemical data fields extracted from the UK Biobank.**

| Data Types | Data Extracted |
| --- | --- |
| Demographic (n=8) | Age, sex, alcohol intake (units/week), smoking history, BMI, waist hip ratio, systolic blood pressure (mmHg), diagnosis of type II diabetes mellitus* |
| Haematological  and  Biochemical (n=65) | ***Haematological***: WBC (x10^9^/L), RBC (x10^9^/L), Haemoglobin Conc. (g/dL), Haematocrit (%), MCV (fL), MCH (pg), MCHC (g/dL), RDW (%), Platelet Count (x10^9^/L), Platelet Crit (%), Platelet Volume (fL), Platelet Distribution Width (%), Lymphocytes (x10^9^/L), Monocytes (x10^9^/L), Neutrophil (x10^9^/L), Eosinophil (x10^9^/L), Basophil (x10^9^/L), Nucleated RBC (x10^9^/L), Lymphocyte Percentage, Monocyte Percentage, Neutrophil Percentage, Eosinophil Percentage, Basophil Percentage, Nucleated RBC Percentage, Reticulocyte Percentage, Reticulocyte Count (x10^12^/L), Mean Reticulocyte Vol. (fL), Mean Spherocyte Vol. (fL), Immature Reticulocyte Fraction (ratio), High Light Scattered Reticulocyte (%), High Light Reticulocyte Count (x10^12^/L).  ***Biochemical***: Urine Microalbumin (mg/L), Urine Creatinine (umol/L), Urine Potassium (mmol/L), Urine Sodium (mmol/L), Albumin (g/dL), ALP (U/L), ALT (U/L), Apolipoprotein A (g/L), Apolipoprotein B (g/L), AST (U/L), Direct Bilirubin (umol/L), Urea (mmol/L), Calcium (mmol/L), Cholesterol (mmol/L), Creatinine (umol/L), CRP (g/L), Cystatin C (mg/L), GGT (U/L), Glucose (mmol/L), HBA1C (mmol/mol), HDL-C (mmol/L), IGF1 (nmol/L), LDL (mmol/L), Lipoprotein A (nmol/L), Oestradiol (pmol/L), Phosphate (mmol/L), Rheumatoid Factor (IU/mL), SHBG (nmol/L), Total Bilirubin (umol/L), Testosterone (nmol/L), Total Protein (g/L), Triglyceride (mmol/L), Urate (umol/L), Vitamin D (nmol/L) |
| Genetics (n=7) | PNPLA3 rs738409, TM6SF2 rs5859426, GCKR rs1260326, HSD17B13 rs72613567 and TRIB1 rs112875651 |

Data extracted are shown from the UK Biobank dataset, which were used in the multiple imputation model and downstream machine learning algorithm training/analysis. *Type 2 diabetes was defined by either elevated HBA1c level measured (>48mmol/mol), a record of anti-diabetic drug prescription or an electronic record of diagnosis based on ICD9/10 coding.

**Table S2 – Characteristics of MRI-PDFF cohort.**

| Characteristics | Healthy | *n* | MASLD | *n* |
| --- | --- | --- | --- | --- |
| Age (Years) | 55 (48-61) | 1932 | 55 (49-60.25) | 620 |
| Sex (Male) | 771 (39.9%) | 1932 | 340 (54.8%) | 620 |
| Smoking Status  Ex-smoker  Current smoker | 500 (25.9%)  83 (4.3%) | 1932 | 177 (28.6%)  39 (6.3%) | 620 |
| BMI | 25.08 (23.05-27.7) | 1930 | 28.77 (26.12-32.28) | 620 |
| Waist Hip Ratio | 0.5 (0.46-0.54) | 1931 | 0.56 (0.52-0.6) | 620 |
| Systolic Blood Pressure (mmHg) | 127 (117-139) | 1932 | 134 (123-144) | 618 |
| Diastolic Blood Pressure (mmHg) | 77 (71-84) | 1932 | 82 (76-88) | 618 |
| Alcohol (units/week) | 8.2 (4.8-11.9) | 1445 | 8.6 (4.5-12.2) | 450 |
| Townsend Deprivation Index | -2.91 (-4.03--1) | 1927 | -2.62 (-3.96--0.3) | 618 |
| Serum |  |  |  |  |
| Direct Bilirubin (µmol/L) | 1.59 (1.28-2.07) | 1573 | 1.6 (1.3-2.03) | 500 |
| AST (U/L) | 23.6 (20.33-27.1) | 1850 | 25.65 (21.7-30.8) | 582 |
| ALT (U/L) | 17.94 (14.24-23.49) | 1857 | 24.88 (19.28-34.32) | 585 |
| ALP | 76.8 (63.9-91.4) | 1857 | 80.3 (68.9-95.5) | 585 |
| GGT (U/L) | 21 (15.7-30.1) | 1856 | 30.7 (21.8-44.4) | 585 |
| Albumin (g/dL) | 45.28 (43.52-46.93) | 1694 | 45.28 (43.55-47.05) | 538 |
| Apolipoprotein A (g/L) | 1.52 (1.35-1.7) | 1687 | 1.4 (1.26-1.54) | 538 |
| Apolipoprotein B (g/L) | 1.01 (0.85-1.17) | 1849 | 1.06 (0.9-1.22) | 580 |
| HBA1C (mmol/mol) | 34.4 (32.2-36.7) | 1841 | 36.1 (33.2-38.63) | 596 |
| Platelets (10^9^ cells/L) | 246.35 (213.3-282.15) | 1882 | 254 (213.15-288.2) | 599 |
| HDL-C (mmol/L) | 1.44 (1.23-1.7) | 1696 | 1.22 (1.05-1.41) | 538 |
| LDL-C (mmol/L) | 3.52 (2.99-4.12) | 1855 | 3.61 (3.04-4.22) | 584 |
| Cholesterol (mmol/L) | 5.64 (4.93-6.44) | 1856 | 5.64 (4.86-6.39) | 585 |
| Triglyceride (mmol/L) | 1.3 (0.94-1.89) | 1854 | 1.82 (1.37-2.53) | 585 |
| Lipoprotein-A (nmol/L) | 21.81 (10.03-60.08) | 1498 | 20.2 (9.04-53.39) | 467 |
| IGF1 (nmol/L) | 22.3 (18.63-25.44) | 1849 | 22.24 (17.93-25.58) | 581 |
| Urate ((µmol/L) | 275.4 (229.1-328.8) | 1849 | 332.4 (281.1-382.1) | 585 |

Table shows the characteristics of the cohort prior to imputation whereby MASLD is defined as MRI-PDFF >5%. Continuous variables are shown as median and interquartile range, whilst categorical data are shown in frequency (%). BMI – body mass index, ALT – alanine aminotransferase, AST – aspartate aminotransferase, GGT – gamma-glutamyl transpeptidase, LDL-C – low density lipoprotein cholesterol, HDL-C - high density lipoprotein cholesterol.

**Table S3 – Hyperparameter selection through grid search of trained machine learning models.**

| **Machine Learning Model (Ground Truth Definition)** | **Trained hyperparameters** |
| --- | --- |
| ***Demographic Features*** |  |
| PLS-DA (MRI-EHR) | Components = 14 |
| PLS-DA (MRI) | Components = 16 |
| Random Forest (MRI-EHR) | Mtry = 6, node size = 1 |
| Random Forest (MRI) | Mtry = 6, node size = 1 |
| ***Haematological and Biochemical Features*** |  |
| PLS-DA (MRI-EHR) | Components = 11 |
| PLS-DA (MRI) | Components = 15 |
| Random Forest (MRI-EH) | Mtry = 4, node size = 2 |
| Random Forest (MRI) | Mtry = 2, node size = 1 |
| ***Metabolome Features*** |  |
| PLS-DA (MRI-EHR) | Components = 176 |
| PLS-DA (MRI) | Components = 172 |
| Random Forest (MRI-EHR) | Mtry = 1, node size = 1 |
| Random Forest (MRI) | Mtry = 1, node size = 1 |
| ***Demographic + Haematological and Biochemical Features*** |  |
| PLS-DA (MRI-EHR) | Components = 8 |
| PLS-DA (MRI) | Components = 12 |
| Random Forest (MRI-EHR) | Mtry = 10, node = 1 |
| Random Forest (MRI) | Mtry = 3, node = 1 |
| Logistic regression (MRI-EHR) | N/A |
| Logistic regression (MRI) | N/A |
| Support Vector Machine – Linear Kernel (MRI-EHR) | C=0.3 |
| Support Vector Machine – Linear Kernel (MRI) | C=4.4 |
| ***All Selected Features*** |  |
| PLS-DA (MRI-EHR) | Components = 1 |
| PLS-DA (MRI) | Components = 1 |
| Random Forest (MRI-EHR) | Mtry = 10, node size = 2 |
| Random Forest (MRI) | Mtry = 1, node size = 1 |
| Support Vector Machine – Linear Kernel (MRI-EHR) | C=0.5 |
| Support Vector Machine – Linear Kernel (MRI) | C=2.6 |
| Logistic regression (MRI-EHR) | N/A |
| Logistic regression (MRI) | N/A |

Table above shows machine learning models trained against different ground truth definitions of MASLD. The hyperparameter values were obtained by grid search. MRI-EHR denotes ground truth case definition derived from MRI-PDFF and electronic health record diagnostic codes and MRI denotes derivation from MRI-PDFF alone.

**Table S4 – Positive and Negative Predictive Value Across Prevalence Ranges of MASLD (DHB Feature models)**

|  | Logistic Regression | | Partial Least Square - DA | | Support Vector Machine - LK | | Random Forest | |
| --- | --- | --- | --- | --- | --- | --- | --- | --- |
|  | MRI cohort | | | | | | | |
| Prevalence | PPV (95% CI) | NPV (95% CI) | PPV (95% CI) | NPV (95% CI) | PPV (95% CI) | NPV (95% CI) | PPV (95% CI) | NPV (95% CI) |
| 0.01 | 1.00 (1.00-1.00) | 0.02 (0.02-0.02) | 1.00 (1.00 -1.00) | 0.02 (0.02-0.03) | 1.00 (1.00-1.00) | 0.02 (0.02-0.03) | 1.00 (1.00-1.00) | 0.03 (0.02-0.03) |
| 0.05 | 0.99 (0.98-0.99) | 0.10 (0.09-0.12) | 0.98 (0.98-0.99) | 0.11 (0.10-0.12) | 0.98 (0.98-0.99) | 0.11 (0.10-0.13) | 0.98 (0.98-0.99) | 0.12 (0.11-0.13) |
| 0.10 | 0.97 (0.96-0.98) | 0.20 (0.18-0.22) | 0.97 (0.96-0.98) | 0.21 (0.19-0.23) | 0.97 (0.96-0.97) | 0.22 (0.19-0.24) | 0.97 (0.96-0.97) | 0.22 (0.20-0.25) |
| 0.15 | 0.95 (0.94-0.97) | 0.28 (0.26-0.30) | 0.95 (0.94-0.97) | 0.29 (0.27-0.32) | 0.95 (0.93-0.96) | 0.30 (0.28-0.33) | 0.95 (0.93-0.96) | 0.31 (0.29-0.34) |
| 0.20 | 0.94 (0.92-0.95) | 0.36 (0.33-0.38) | 0.93 (0.91-0.95) | 0.37 (0.34-0.40) | 0.93 (0.91-0.94) | 0.38 (0.35-0.41) | 0.93 (0.91-0.94) | 0.39 (0.36-0.43) |
| 0.25 | 0.92 (0.90-0.94) | 0.43 (0.40-0.45) | 0.91 (0.88-0.94) | 0.44 (0.41-0.47) | 0.90 (0.88-0.93) | 0.45 (0.42-0.48) | 0.90 (0.88-0.92) | 0.46 (0.43-0.50) |
| 0.30 | 0.90 (0.87-0.92) | 0.49 (0.46-0.52) | 0.89 (0.86-0.92) | 0.50 (0.47-0.53) | 0.88 (0.85-0.91) | 0.51 (0.48-0.55) | 0.88 (0.85-0.9) | 0.53 (0.49-0.56) |
| 0.35 | 0.87 (0.84-0.91) | 0.54 (0.52-0.57) | 0.86 (0.82-0.90) | 0.56 (0.53-0.59) | 0.85 (0.82-0.88) | 0.57 (0.54-0.60) | 0.85 (0.82-0.88) | 0.58 (0.55-0.62) |
| 0.40 | 0.85 (0.81-0.89) | 0.60 (0.57-0.62) | 0.84 (0.79-0.88) | 0.61 (0.58-0.64) | 0.82 (0.79-0.86) | 0.62 (0.59-0.65) | 0.82 (0.79-0.86) | 0.63 (0.60-0.66) |
| 0.45 | 0.82 (0.78-0.86) | 0.65 (0.62-0.67) | 0.81 (0.76-0.86) | 0.66 (0.63-0.69) | 0.79 (0.75-0.83) | 0.67 (0.64-0.70) | 0.79 (0.75-0.83) | 0.68 (0.65-0.71) |
| 0.50 | 0.79 (0.74-0.84) | 0.69 (0.67-0.71) | 0.77 (0.72-0.83) | 0.70 (0.68-0.73) | 0.76 (0.71-0.80) | 0.71 (0.69-0.74) | 0.76 (0.71-0.80) | 0.72 (0.69-0.75) |
|  | MRI-EHR Cohort | | | | | | | |
| 0.01 | 1.00 (1.00-1.00) | 0.04 (0.03-0.05) | 1.00 (1.00-1.00) | 0.04 (0.03-0.04) | 1.00 (1.00-1.00) | 0.04 (0.03-0.05) | 1.00 (1.00-1.00) | 0.04 (0.02-0.06) |
| 0.05 | 0.99 (0.98-0.99) | 0.17 (0.15-0.20) | 0.99 (0.98-0.99) | 0.16 (0.14-0.18) | 0.99 (0.98-0.99) | 0.18 (0.14-0.22) | 0.99 (0.98-0.99) | 0.17 (0.10-0.24) |
| 0.10 | 0.97 (0.97-0.98) | 0.31 (0.27-0.34) | 0.97 (0.97-0.98) | 0.29 (0.26-0.32) | 0.97 (0.96-0.98) | 0.32 (0.26-0.38) | 0.97 (0.96-0.98) | 0.30 (0.20-0.40) |
| 0.15 | 0.96 (0.95-0.96) | 0.41 (0.37-0.46) | 0.96 (0.95-0.96) | 0.40 (0.36-0.43) | 0.96 (0.94-0.97) | 0.43 (0.36-0.49) | 0.95 (0.94-0.97) | 0.40 (0.29-0.51) |
| 0.20 | 0.94 (0.93-0.95) | 0.50 (0.45-0.54) | 0.94 (0.93-0.95) | 0.48 (0.45-0.52) | 0.94 (0.92-0.95) | 0.51 (0.45-0.58) | 0.94 (0.91-0.96) | 0.49 (0.38-0.60) |
| 0.25 | 0.92 (0.91-0.93) | 0.57 (0.52-0.61) | 0.92 (0.91-0.93) | 0.55 (0.52-0.59) | 0.92 (0.90-0.94) | 0.59 (0.52-0.65) | 0.92 (0.89-0.94) | 0.56 (0.45-0.67) |
| 0.30 | 0.90 (0.89-0.92) | 0.63 (0.59-0.67) | 0.90 (0.89-0.91) | 0.61 (0.58-0.65) | 0.90 (0.88-0.92) | 0.64 (0.58-0.71) | 0.90 (0.86-0.93) | 0.62 (0.52-0.72) |
| 0.35 | 0.88 (0.86-0.90) | 0.68 (0.64-0.72) | 0.88 (0.86-0.89) | 0.67 (0.64-0.70) | 0.88 (0.85-0.90) | 0.70 (0.64-0.75) | 0.87 (0.83-0.91) | 0.67 (0.58-0.77) |
| 0.40 | 0.86 (0.84-0.88) | 0.73 (0.69-0.76) | 0.85 (0.84-0.87) | 0.71 (0.68-0.74) | 0.85 (0.82-0.88) | 0.74 (0.69-0.79) | 0.85 (0.80-0.89) | 0.72 (0.63-0.80) |
| 0.45 | 0.83 (0.81-0.85) | 0.76 (0.73-0.80) | 0.83 (0.81-0.85) | 0.75 (0.73-0.78) | 0.82 (0.79-0.86) | 0.78 (0.73-0.82) | 0.82 (0.76-0.87) | 0.76 (0.68-0.83) |
| 0.50 | 0.80 (0.77-0.82) | 0.80 (0.77-0.83) | 0.80 (0.77-0.82) | 0.79 (0.76-0.81) | 0.79 (0.75-0.84) | 0.81 (0.77-0.85) | 0.79 (0.73-0.84) | 0.79 (0.72-0.86) |

Positive and Negative predictive values shown for learning models using selected demographic, haematological and biochemical (DHB) features on the validation set. Mean and 95% CI shown derived using Rubin’s Rules across imputed validation sets and confidence interval calculated from bootstrapping.

**Table S5 - Positive and Negative Predictive Value Across Prevalence Ranges of MASLD (All Feature Models)**

|  | Logistic Regression | | Partial Least Square - DA | | Support Vector Machine - LK | | Random Forest | |
| --- | --- | --- | --- | --- | --- | --- | --- | --- |
|  | MRI cohort | | | | | | | |
| Prevalence | PPV (95% CI) | NPV (95% CI) | PPV (95% CI) | NPV (95% CI) | PPV (95% CI) | NPV (95% CI) | PPV (95% CI) | NPV (95% CI) |
| 0.01 | 1.00 (1.00-1.00) | 0.03 (0.02-0.04) | 0.99 (0.99-1.00) | 0.04 (0.03-0.05) | 1.00 (1.00 -1.00) | 0.02 (0.02-0.03) | 1.00 (1.00 -1.00) | 0.02 (0.02-0.03) |
| 0.05 | 0.98 (0.98-0.98) | 0.15 (0.12-0.18) | 0.97 (0.97-0.98) | 0.17 (0.14-0.20) | 0.99 (0.98-0.99) | 0.11 (0.10-0.12) | 0.98 (0.98-0.99) | 0.11 (0.10-0.12) |
| 0.10 | 0.96 (0.95-0.97) | 0.27 (0.22-0.32) | 0.94 (0.93-0.95) | 0.30 (0.25-0.35) | 0.98 (0.97-0.98) | 0.21 (0.19-0.23) | 0.97 (0.96-0.98) | 0.21 (0.19-0.23) |
| 0.15 | 0.93 (0.92-0.95) | 0.37 (0.31-0.42) | 0.91 (0.9-0.92) | 0.41 (0.35-0.46) | 0.96 (0.95-0.97) | 0.29 (0.27-0.32) | 0.95 (0.94-0.97) | 0.30 (0.27-0.32) |
| 0.20 | 0.91 (0.89-0.93) | 0.45 (0.39-0.51) | 0.88 (0.86-0.89) | 0.49 (0.44-0.55) | 0.95 (0.93-0.96) | 0.37 (0.35-0.40) | 0.93 (0.91-0.95) | 0.37 (0.34-0.40) |
| 0.25 | 0.88 (0.86-0.90) | 0.52 (0.47-0.58) | 0.84 (0.83-0.86) | 0.56 (0.51-0.62) | 0.93 (0.91-0.95) | 0.44 (0.41-0.47) | 0.91 (0.89-0.94) | 0.44 (0.41-0.47) |
| 0.30 | 0.85 (0.83-0.88) | 0.59 (0.53-0.64) | 0.81 (0.79-0.83) | 0.62 (0.57-0.67) | 0.91 (0.89-0.94) | 0.50 (0.48-0.53) | 0.89 (0.86-0.92) | 0.50 (0.47-0.54) |
| 0.35 | 0.82 (0.79-0.85) | 0.64 (0.59-0.69) | 0.77 (0.75-0.79) | 0.67 (0.63-0.72) | 0.89 (0.86-0.92) | 0.56 (0.53-0.59) | 0.86 (0.83-0.90) | 0.56 (0.53-0.59) |
| 0.40 | 0.79 (0.75-0.82) | 0.69 (0.64-0.74) | 0.73 (0.7-0.76) | 0.72 (0.68-0.76) | 0.87 (0.84-0.91) | 0.61 (0.59-0.64) | 0.84 (0.79-0.88) | 0.61 (0.58-0.64) |
| 0.45 | 0.75 (0.71-0.79) | 0.73 (0.68-0.78) | 0.69 (0.66-0.72) | 0.76 (0.72-0.80) | 0.85 (0.81-0.89) | 0.66 (0.63-0.68) | 0.81 (0.76-0.86) | 0.66 (0.63-0.69) |
| 0.50 | 0.71 (0.67-0.76) | 0.77 (0.73-0.81) | 0.64 (0.61-0.67) | 0.79 (0.76-0.83) | 0.82 (0.77-0.87) | 0.70 (0.68-0.73) | 0.77 (0.72-0.83) | 0.70 (0.68-0.73) |
|  | MRI-EHR Cohort | | | | | | | |
| 0.01 | 1.00 (1.00 -1.00) | 0.05 (0.04-0.06) | 1.00 (1.00 -1.00) | 0.05 (0.04-0.06) | 1.00 (1.00 -1.00) | 0.05 (0.03-0.07) | 1.00 (1.00-1.00) | 0.03 (0.03-0.04) |
| 0.05 | 0.98 (0.98-0.99) | 0.22 (0.18-0.25) | 0.98 (0.98-0.99) | 0.22 (0.18-0.25) | 0.98 (0.98-0.99) | 0.23 (0.16-0.29) | 0.99 (0.98-0.99) | 0.16 (0.14-0.18) |
| 0.10 | 0.97 (0.96-0.97) | 0.37 (0.32-0.42) | 0.97 (0.96-0.97) | 0.37 (0.32-0.42) | 0.97 (0.96-0.97) | 0.38 (0.29-0.47) | 0.97 (0.97-0.98) | 0.28 (0.25-0.31) |
| 0.15 | 0.95 (0.94-0.96) | 0.48 (0.42-0.53) | 0.95 (0.94-0.96) | 0.48 (0.43-0.53) | 0.95 (0.94-0.96) | 0.49 (0.40-0.59) | 0.96 (0.95-0.96) | 0.38 (0.35-0.42) |
| 0.20 | 0.93 (0.92-0.94) | 0.57 (0.51-0.62) | 0.93 (0.92-0.94) | 0.57 (0.51-0.62) | 0.93 (0.92-0.94) | 0.58 (0.49-0.67) | 0.94 (0.93-0.95) | 0.47 (0.43-0.50) |
| 0.25 | 0.91 (0.90-0.92) | 0.63 (0.58-0.68) | 0.91 (0.90-0.92) | 0.64 (0.59-0.69) | 0.91 (0.89-0.92) | 0.65 (0.56-0.73) | 0.92 (0.91-0.93) | 0.54 (0.51-0.58) |
| 0.30 | 0.89 (0.87-0.90) | 0.69 (0.64-0.74) | 0.88 (0.87-0.90) | 0.69 (0.65-0.74) | 0.89 (0.87-0.90) | 0.70 (0.62-0.78) | 0.90 (0.88-0.91) | 0.60 (0.57-0.64) |
| 0.35 | 0.86 (0.84-0.88) | 0.74 (0.69-0.78) | 0.86 (0.84-0.88) | 0.74 (0.70-0.78) | 0.86 (0.84-0.88) | 0.75 (0.67-0.82) | 0.88 (0.86-0.89) | 0.66 (0.62-0.69) |
| 0.40 | 0.83 (0.81-0.85) | 0.78 (0.74-0.81) | 0.83 (0.81-0.85) | 0.78 (0.74-0.81) | 0.83 (0.81-0.86) | 0.78 (0.72-0.85) | 0.85 (0.83-0.87) | 0.70 (0.67-0.73) |
| 0.45 | 0.80 (0.78-0.83) | 0.81 (0.78-0.84) | 0.80 (0.78-0.82) | 0.81 (0.78-0.84) | 0.80(0.77-0.83) | 0.82 (0.76-0.87) | 0.82 (0.80-0.85) | 0.74 (0.72-0.77) |
| 0.50 | 0.77 (0.75-0.79) | 0.84 (0.81-0.87) | 0.77 (0.74-0.79) | 0.84 (0.81-0.87) | 0.77 (0.73-0.80) | 0.85 (0.80-0.90) | 0.79 (0.77-0.82) | 0.78 (0.76-0.80) |

Positive and Negative predictive values shown for learning models using all selected features on the validation set. Mean and 95% CI shown derived using Rubin’s Rules across imputed validation sets and confidence interval calculated from bootstrapping.

**Table S6 – F1 Scores of Models Across Prevalence Ranges of MASLD (DHB Feature Models)**

|  | Logistic Regression | Partial Least Square - DA | Support Vector Machine - LK | Random Forest |
| --- | --- | --- | --- | --- |
|  | MRI cohort | | | |
| Prevalence | F1 (95% CI) | F1 (95% CI) | F1 (95% CI) | F1 (95% CI) |
| 0.01 | 0.77 (0.74-0.80) | 0.79 (0.76-0.83) | 0.81 (0.78-0.83) | 0.82 (0.80-0.85) |
| 0.05 | 0.77 (0.74-0.79) | 0.79 (0.75-0.82) | 0.81 (0.78-0.83) | 0.82 (0.80-0.84) |
| 0.1 | 0.76 (0.73-0.79) | 0.78 (0.75-0.82) | 0.80(0.77-0.82) | 0.81 (0.79-0.84) |
| 0.15 | 0.76 (0.73-0.78) | 0.78 (0.74-0.81) | 0.79 (0.77-0.82) | 0.81 (0.78-0.83) |
| 0.2 | 0.75 (0.72-0.78) | 0.77 (0.74-0.80) | 0.79 (0.76-0.81) | 0.80 (0.77-0.82) |
| 0.25 | 0.74 (0.72-0.77) | 0.76 (0.73-0.80) | 0.78 (0.75-0.80) | 0.79 (0.76-0.81) |
| 0.3 | 0.74 (0.71-0.76) | 0.76 (0.72-0.79) | 0.77 (0.74-0.79) | 0.78 (0.76-0.81) |
| 0.35 | 0.73 (0.70-0.76) | 0.75 (0.72-0.78) | 0.76 (0.73-0.78) | 0.77 (0.74-0.80) |
| 0.4 | 0.72 (0.69-0.75) | 0.74 (0.71-0.77) | 0.75 (0.72-0.77) | 0.76 (0.73-0.78) |
| 0.45 | 0.71 (0.68-0.74) | 0.72 (0.69-0.75) | 0.73 (0.70-0.76) | 0.74 (0.72-0.77) |
| 0.5 | 0.70 (0.67-0.73) | 0.71 (0.68-0.74) | 0.72 (0.69-0.75) | 0.73 (0.70-0.76) |
|  | MRI-EHR Cohort | | | |
| 0.01 | 0.89 (0.86-0.91) | 0.88 (0.86-0.90) | 0.90 (0.86-0.93) | 0.88 (0.82-0.94) |
| 0.05 | 0.88 (0.86-0.91) | 0.87 (0.86-0.89) | 0.89 (0.85-0.93) | 0.88 (0.82-0.94) |
| 0.1 | 0.88 (0.85-0.90) | 0.87 (0.85-0.89) | 0.89 (0.85-0.92) | 0.87 (0.81-0.93) |
| 0.15 | 0.87 (0.85-0.89) | 0.86 (0.84-0.88) | 0.88 (0.84-0.91) | 0.86 (0.81-0.92) |
| 0.2 | 0.86 (0.84-0.89) | 0.86 (0.84-0.87) | 0.87 (0.84-0.90) | 0.86 (0.81-0.91) |
| 0.25 | 0.86 (0.83-0.88) | 0.85 (0.83-0.87) | 0.86 (0.83-0.89) | 0.85 (0.80-0.89) |
| 0.3 | 0.85 (0.82-0.87) | 0.84 (0.82-0.86) | 0.85 (0.82-0.88) | 0.84 (0.80-0.88) |
| 0.35 | 0.84 (0.81-0.86) | 0.83 (0.81-0.85) | 0.84 (0.82-0.87) | 0.83 (0.79-0.87) |
| 0.4 | 0.83 (0.80-0.85) | 0.82 (0.80-0.84) | 0.83 (0.81-0.86) | 0.82 (0.78-0.85) |
| 0.45 | 0.81 (0.79-0.84) | 0.81 (0.78-0.83) | 0.82 (0.79-0.84) | 0.80 (0.77-0.83) |
| 0.5 | 0.80 (0.78-0.82) | 0.79 (0.77-0.81) | 0.80 (0.78-0.82) | 0.79 (0.76-0.81) |

F1 score and 95%CI for models shown for models trained with selected demographic, haematological and biochemical (DHB) features. Score statistics were derived from imputed test sets and combined using Rubin’s Rules. Prevalence was simulated via case weighting.

**Table S7 - F1 Scores of Models Across Prevalence Ranges of MASLD (All Feature Models)**

|  | Logistic Regression | Partial Least Square - DA | Support Vector Machine - LK | Random Forest |
| --- | --- | --- | --- | --- |
|  | MRI cohort | | | |
| Prevalence | F1 (95% CI) | F1 (95% CI) | F1 (95% CI) | F1 (95% CI) |
| 0.01 | 0.88 (0.85-0.92) | 0.93 (0.91-0.94) | 0.78 (0.75-0.80) | 0.79 (0.76-0.83) |
| 0.05 | 0.88 (0.84-0.91) | 0.92 (0.90-0.93) | 0.77 (0.75-0.80) | 0.79 (0.75-0.83) |
| 0.1 | 0.87 (0.83-0.90) | 0.90 (0.89-0.92) | 0.77 (0.74-0.80) | 0.78 (0.75-0.82) |
| 0.15 | 0.86 (0.83-0.89) | 0.89 (0.87-0.90) | 0.77 (0.74-0.79) | 0.78 (0.74-0.81) |
| 0.2 | 0.85 (0.82-0.88) | 0.87 (0.86-0.89) | 0.76 (0.74-0.79) | 0.77 (0.74-0.81) |
| 0.25 | 0.83 (0.80-0.86) | 0.85 (0.84-0.87) | 0.76 (0.73-0.78) | 0.76 (0.73-0.80) |
| 0.3 | 0.82 (0.79-0.85) | 0.84 (0.82-0.85) | 0.75 (0.72-0.78) | 0.76 (0.72-0.79) |
| 0.35 | 0.81 (0.78-0.84) | 0.81 (0.80-0.83) | 0.74 (0.72-0.77) | 0.75 (0.72-0.78) |
| 0.4 | 0.79 (0.76-0.82) | 0.79 (0.77-0.81) | 0.74 (0.71-0.76) | 0.74 (0.71-0.77) |
| 0.45 | 0.77 (0.74-0.80) | 0.77 (0.74-0.79) | 0.73 (0.70-0.76) | 0.73 (0.69-0.76) |
| 0.5 | 0.75 (0.72-0.78) | 0.74 (0.71-0.76) | 0.72 (0.69-0.75) | 0.71 (0.68-0.74) |
|  | MRI-EHR Cohort | | | |
| 0.01 | 0.92 (0.90-0.94) | 0.92 (0.9-0.94) | 0.93 (0.89-0.96) | 0.87 (0.85-0.89) |
| 0.05 | 0.92 (0.90-0.93) | 0.92 (0.9-0.94) | 0.92 (0.89-0.95) | 0.87 (0.85-0.89) |
| 0.1 | 0.91 (0.89-0.93) | 0.91 (0.89-0.93) | 0.91 (0.88-0.94) | 0.86 (0.84-0.88) |
| 0.15 | 0.90 (0.88-0.92) | 0.90 (0.88-0.92) | 0.9 (0.87-0.93) | 0.86 (0.84-0.88) |
| 0.2 | 0.89 (0.87-0.91) | 0.89 (0.87-0.91) | 0.9 (0.87-0.92) | 0.85 (0.83-0.87) |
| 0.25 | 0.88 (0.86-0.90) | 0.88 (0.86-0.90) | 0.89 (0.86-0.91) | 0.84 (0.82-0.86) |
| 0.3 | 0.87 (0.85-0.89) | 0.87 (0.85-0.89) | 0.87 (0.85-0.90) | 0.83 (0.81-0.85) |
| 0.35 | 0.86 (0.84-0.88) | 0.86 (0.84-0.88) | 0.86 (0.84-0.89) | 0.82 (0.80-0.84) |
| 0.4 | 0.84 (0.83-0.86) | 0.84 (0.83-0.86) | 0.85 (0.82-0.87) | 0.81 (0.79-0.83) |
| 0.45 | 0.83 (0.81-0.85) | 0.83 (0.81-0.85) | 0.83 (0.81-0.85) | 0.80 (0.78-0.82) |
| 0.5 | 0.81 (0.79-0.83) | 0.81 (0.79-0.83) | 0.81 (0.79-0.83) | 0.78 (0.76-0.81) |

F1 score and 95%CI for models shown for models trained with all selected features (demographic, haematological, biochemical and metabolome). Score statistics were derived from imputed test sets and combined using Rubin’s Rules. Prevalence was simulated via case weighting.

**Table S8 – Predictors selected by stepwise logistic regression**

|  | Demographic, Haematological and Biochemical Features | All Features |
| --- | --- | --- |
| MRI Cohort | Age | Cholesterol in Medium LDL |
|  | BMI | Cholesterol in Very Large VLDL |
|  | Waist Hip Ratio | Cholesterol to Total Lipids in Large HDL percentage |
|  | Systolic Blood Pressure | Cholesterol to Total Lipids in Large LDL percentage |
|  | Immature Reticulocyte Fraction | Cholesterol to Total Lipids in Very Small VLDL percentage |
|  | Urine Sodium | Cholesteryl Esters to Total Lipids in Large LDL percentage |
|  | ALT | Glycoprotein Acetyls |
|  | ApoA | Free Cholesterol to Total Lipids in Large HDL percentage |
|  | CRP | Linoleic Acid to Total Fatty Acids percentage |
|  | GGT | Omega-6 Fatty Acids to Total Fatty Acids percentage |
|  | HDL-C | Phospholipids in LDL |
|  | SHBG | Phospholipids to Total Lipids in Large HDL percentage |
|  | Triglyceride | Polyunsaturated Fatty Acids to Monounsaturated Fatty Acids ratio |
|  | Urate | Polyunsaturated Fatty Acids to Total Fatty Acids percentage |
|  | Sex | Total Concentration of Branched-Chain Amino Acids (Leucine + Isoleucine + Valine) |
|  |  | Total Lipids in Large HDL |
|  |  | Total Lipids in LDL |
|  |  | Total Lipids in Medium LDL |
|  |  | Total Lipids in Small LDL |
|  |  | Total Lipids in Small HDL |
|  |  | Triglycerides in Large LDL |
|  |  | Triglycerides in LDL |
|  |  | Triglycerides to Total Lipids in Large LDL percentage |
|  |  | Triglycerides to Total Lipids in Very Small VLDL percentage |
|  |  | VLDL Cholesterol |
|  |  | ALT |
|  |  | HDL-C |
|  |  | SHBG |
|  |  | Urate |
|  |  | CRP |
|  |  | Immature Reticulocyte Fraction |
|  |  | BMI |
|  |  | Waist Hip Ratio |
|  |  | Age |
|  |  | Sex |
|  |  | Systolic Blood Pressure |
| MRI - EHR Cohort | Age | Cholesterol in Medium LDL |
|  | BMI | Cholesterol in Small LDL |
|  | Waist Hip Ratio | Cholesterol in Very Large VLDL |
|  | Systolic Blood Pressure | Cholesterol to Total Lipids in Large LDL percentage |
|  | Townsend Deprivation Index | Cholesterol to Total Lipids in Very Small VLDL percentage |
|  | High Light Scattered Reticulocyte | Cholesteryl Esters in Very Large HDL |
|  | Urine Sodium | Cholesteryl Esters to Total Lipids in Large LDL percentage |
|  | ALT | Glycoprotein Acetyls |
|  | Apolipoprotein A | Linoleic Acid to Total Fatty Acids percentage |
|  | CRP | Omega-6 Fatty Acids to Total Fatty Acids percentage |
|  | GGT | Phospholipids in LDL |
|  | HBA1c | Phospholipids in Very Large HDL |
|  | HDL-C | Polyunsaturated Fatty Acids to Total Fatty Acids percentage |
|  | SHBG | Total Concentration of Branched-Chain Amino Acids (Leucine + Isoleucine + Valine) |
|  | Triglyceride | Total Lipids in Large HDL |
|  | Urate | Total Lipids in Medium LDL |
|  | Sex | Total Lipids in Small HDL |
|  |  | Total Lipids in Very Small VLDL |
|  |  | Triglycerides in Large LDL |
|  |  | Triglycerides in LDL |
|  |  | Triglycerides to Total Lipids in Large LDL percentage |
|  |  | Triglycerides to Total Lipids in Very Small VLDL percentage |
|  |  | VLDL Cholesterol |
|  |  | GGT |
|  |  | ALT |
|  |  | High Light Scattered Reticulocte |
|  |  | SHBG |
|  |  | Urate |
|  |  | Apolipoprotein A |
|  |  | CRP |
|  |  | Urine Sodium |
|  |  | HBA1C |
|  |  | BMI |
|  |  | Waist Hip Ratio |
|  |  | Townsend Deprivation Index |
|  |  | Sex |
|  |  | Systolic Blood Pressure |

**Table S9 – Median follow-up period of all disease outcomes of interest**

| Disease outcome | Median follow-up (IQR) (days) |
| --- | --- |
| Ischaemic stroke | 1378 (793-2056) |
| Ischaemic heart disease | 930 (393-2188) |
| Hepatic malignancy | 1038 (301-1231) |
| Extrahepatic malignancy | 989 (494-2019) |
| Hypertension | 1166 (521.5-2296) |
| Dyslipidaemia | 1122 (356-2328) |
| Type 2 diabetes mellitus | 1115 (456-2085) |
| All-cause mortality | 3352 (2286-3948) |

Table shows disease follow-up period (days) overall for MASLD individuals. Disease outcomes were extracted up till December 31^st^, 2019, to avoid diagnostic coding in electronic health records being inaccurate due to onset of the COVID pandemic.

**Figure S1 – Correlation Heatmap of Plasma Metabolome.**

Above shows the correlation between plasma metabolome measurements (n=249) in the first trance of the data release from the UK Biobank.

**Figure S2 - Variable Importance in Projection (Scaled) for Partial Least Squares Regression algorithm using demographic and genetic features.** Shown are top 10 features selected. Top graph shows algorithm trained against MRI-PDFF and electronic health record definition and bottom against MRI-PDFF defined cases alone. wHR – waist hip ratio, BMI – body mass index. “.Q” and “.L” denotes linear and quadratic terms in the model included by the R when models include categorical variables.

**Figure S3 - Variable Importance Plot (Scaled) for Random Forest algorithm using demographic and genetic features.** Shown are top 10 features selected. Top graph shows algorithm trained against MRI-PDFF and electronic health record definition and bottom against MRI-PDFF defined cases alone. wHR – waist hip ratio, BMI – body mass index. “.Q” and “.L” denotes linear and quadratic terms in the model included by the R when models include categorical variables.

**Figure S4 - Receiver operating characteristics curves of partial least squares-discriminant analysis trained with demographics and genetics features to classify MASLD risk (defined by MRI-PDFF alone) across five imputed datasets.** AUC (95% CI) are shown along with best cut off determined by Youden’s index along with respective sensitivity and specificities.

**Figure S5 - Receiver operating characteristics curves of partial least squares-discriminant analysis trained with demographics and genetics features to classify MASLD risk (cases defined by MRI-PDFF and electronic health records) across five imputed datasets.** AUC (95% CI) are shown along with best cut off determined by Youden’s index along with respective sensitivity and specificities.

**Figure S6 - Receiver operating characteristics curves of random forest trained with demographics and genetics features to classify MASLD risk (cases defined by MRI-PDFF alone) across five imputed datasets.** AUC (95% CI) are shown along with best cut off determined by Youden’s index along with respective sensitivity and specificities.

**Figure S7 - Receiver operating characteristics curves of random forest trained with demographics and genetics features to classify MASLD risk (cases defined by MRI-PDFF and electronic health records) across five imputed datasets.** AUC (95% CI) are shown along with best cut off determined by Youden’s index along with respective sensitivity and specificities.

**Figure S8 - Receiver operating characteristics curves of partial least squares-discriminant analysis trained with haematology and biochemistry features to classify MASLD risk (cases defined by MRI-PDFF alone) across five imputed datasets.** AUC (95% CI) are shown along with best cut off determined by Youden’s index along with respective sensitivity and specificities.

**Figure S9 - Receiver operating characteristics curves of partial least squares-discriminant analysis trained with haematology and biochemistry features to classify MASLD risk (cases defined by MRI-PDFF and electronic health records) across five imputed datasets.** AUC (95% CI) are shown along with best cut off determined by Youden’s index along with respective sensitivity and specificities.

**Figure S10 - Receiver operating characteristics curves of random forest trained with haematology and biochemistry features to classify MASLD risk (cases defined by MRI-PDFF alone) across five imputed datasets.** AUC (95% CI) are shown along with best cut off determined by Youden’s index along with respective sensitivity and specificities.

**Figure S11 - Receiver operating characteristics curves of random forest trained with haematology and biochemistry features to classify MASLD risk (cases defined by MRI-PDFF and electronic health records) across five imputed datasets.** AUC (95% CI) are shown along with best cut off determined by Youden’s index along with respective sensitivity and specificities.

**Figure S12 - Variable Importance in Projection (Scaled) for partial least squares algorithms using haematological and biochemical features.** Shown are the top10 clinical biochemistry features selected. The top graph shows algorithm trained against MRI-PDFF MRI-PDFF and electronic health record and bottom graph against cases defined by MRI-PDFF alone. ApoA – Apolipoprotein A, “Hbconc” – haemoglobin concentration, HDL_C – High Density Lipoprotein Cholesterol, “HighLightRetCount” – High Light Reticulocyte Count, HighLightScatRet – High Light Scattered Reticulocytes, “ImmRetFrac” – immature reticulocyte fraction, SHBG – Sex Hormone Binding Globulin, “Urine_Na” – urine sodium.

**Figure S13 - Variable Importance Plot (Scaled) for Random Forest algorithm using haematological and biochemical features.** Shown are the top 10 clinical biochemistry features selected. The top graph shows algorithms trained against MRI-PDFF and electronic health record definition and bottom against MRI-PDFF defined cases alone. ALT – Alanine Aminotransferase, CRP – C-reactive protein, GGT – gamma glutamyl transpeptidase, HDL_C – High Density Lipoprotein Cholesterol, “HighLightRetCount” – High Light Reticulocyte Count, HighLightScatRet – High Light Scattered Reticulocytes, “ImmRetFrac” – immature reticulocyte fraction, “RetPerc” – Reticulocyte Percentage, SHBG – Sex Hormone Binding Globulin, TG - triglyceride.

**Figure S14 - Receiver operating characteristics curves of partial least squares-discriminant analysis trained with metabolome features to classify MASLD risk (cases defined by MRI-PDFF alone) across five imputed datasets.** AUC (95% CI) are shown along with best cut off determined by Youden’s index along with respective sensitivity and specificities.

**Figure S15 - Receiver operating characteristics curves of partial least squares-discriminant analysis trained with metabolome features to classify MASLD risk (cases defined by MRI-PDFF and electronic health records) across five imputed datasets.** AUC (95% CI) are shown along with best cut off determined by Youden’s index along with respective sensitivity and specificities.

**Figure S16 - Receiver operating characteristics curves of random forest trained with metabolome features to classify MASLD risk (cases defined by MRI-PDFF alone) across five imputed datasets.** AUC (95% CI) are shown along with best cut off determined by Youden’s index along with respective sensitivity and specificities.

**Figure S17 - Receiver operating characteristics curves of random forest trained with metabolome features to classify MASLD risk (cases defined by MRI-PDFF and electronic health records) across five imputed datasets.** AUC (95% CI) are shown along with best cut off determined by Youden’s index along with respective sensitivity and specificities.

**Figure S18 - Variable Importance in Projection (Scaled) for Partial Least Squares algorithm using metabolomic features.** Shown are top 10 metabolome features selected. Top graph shows algorithm trained against MRI-PDFF and electronic health record definition and bottom against MRI-PDFF defined cases alone. HDL – High Density Lipoprotein, LDL- Low density lipoprotein.

**Figure S19 - Variable Importance Plot (Scaled) for Random Forest algorithm using metabolomic features.** Shown are top 20 metabolome features selected. Top graph shows algorithm trained against MRI-PDFF and electronic health record definition and bottom against MRI-PDFF defined cases alone. HDL – High Density Lipoprotein, LDL- Low density lipoprotein.

**Figure S20 - Receiver operating characteristics curves of partial least squares-discriminant analysis trained with selected demographics, haematology and biochemistry features to classify MASLD risk (cases defined by MRI-PDFF alone) across five imputed datasets.** AUC (95% CI) are shown along with best cut off determined by Youden’s index along with respective sensitivity and specificities.

**Figure S21 - Receiver operating characteristics curves of random forest trained with demographics, haematology and biochemistry features to classify MASLD risk (cases defined by MRI-PDFF alone) across five imputed datasets.** AUC (95% CI) are shown along with best cut off determined by Youden’s index along with respective sensitivity and specificities.

**Figure S22 - Receiver operating characteristics curves of support vector machine-linear kernel trained with demographics, haematology and biochemistry features to classify MASLD risk (cases defined by MRI-PDFF alone) across five imputed datasets.** AUC (95% CI) are shown along with best cut off determined by Youden’s index along with respective sensitivity and specificities.

**Figure S23 - Receiver operating characteristics curves of logistic regression trained with demographics, haematology and biochemistry features to classify MASLD risk (cases defined by MRI-PDFF alone) across five imputed datasets.** AUC (95% CI) are shown along with best cut off determined by Youden’s index along with respective sensitivity and specificities.

**Figure S24 - Receiver operating characteristics curves of partial least squares-discriminant analysis trained with demographics, haematology and biochemistry features to classify MASLD risk (cases defined by MRI-PDFF and electronic health records) across five imputed datasets.** AUC (95% CI) are shown along with best cut off determined by Youden’s index along with respective sensitivity and specificities.

**Figure S25 - Receiver operating characteristics curves of random forest discriminant analysis trained with demographics, haematology and biochemistry features to classify MASLD risk (cases defined by MRI-PDFF and electronic health records) across five imputed datasets.** AUC (95% CI) are shown along with best cut off determined by Youden’s index along with respective sensitivity and specificities.

**Figure S26 - Receiver operating characteristics curves of support vector machine linear kernel trained with demographics, haematology and biochemistry features to classify MASLD risk (cases defined by MRI-PDFF and electronic health records) across five imputed datasets.** AUC (95% CI) are shown along with best cut off determined by Youden’s index along with respective sensitivity and specificities.

**Figure S27 - Receiver operating characteristics curves of logistic regression trained with demographics, haematology and biochemistry features to classify MASLD risk (cases defined by MRI-PDFF and electronic health records) across five imputed datasets.** AUC (95% CI) are shown along with best cut off determined by Youden’s index along with respective sensitivity and specificities.

**Figure S28 - Receiver operating characteristics curves of partial least squares-discriminant analysis trained with demographics, haematology and biochemistry and metabolome features to classify MASLD risk (cases defined by MRI-PDFF alone) across five imputed datasets.** AUC (95% CI) are shown along with best cut off determined by Youden’s index along with respective sensitivity and specificities.

**Figure S29 - Receiver operating characteristics curves of random forest trained with demographics, haematology and biochemistry and metabolome features to classify MASLD risk (cases defined by MRI-PDFF alone) across five imputed datasets.** AUC (95% CI) are shown along with best cut off determined by Youden’s index along with respective sensitivity and specificities.

**Figure S30 - Receiver operating characteristics curves of support vector machine linear kernel trained with demographics, haematology and biochemistry and metabolome features to classify MASLD risk (cases defined by MRI-PDFF alone) across five imputed datasets.** AUC (95% CI) are shown along with best cut off determined by Youden’s index along with respective sensitivity and specificities.

**Figure S31 - Receiver operating characteristics curves of logistic regression trained with demographics, haematology and biochemistry and metabolome features to classify MASLD risk (cases defined by MRI-PDFF alone) across five imputed datasets.** AUC (95% CI) are shown along with best cut off determined by Youden’s index along with respective sensitivity and specificities.

**Figure S32 - Receiver operating characteristics curves of partial least squares-discriminant analysis trained with demographics, haematology and biochemistry and metabolome features to classify MASLD risk (cases defined by MRI-PDFF electronic health records) across five imputed datasets.** AUC (95% CI) are shown along with best cut off determined by Youden’s index along with respective sensitivity and specificities.

**Figure S33 - Receiver operating characteristics curves of random forest trained with demographics, haematology and biochemistry and metabolome features to classify MASLD risk (cases defined by MRI-PDFF electronic health records) across five imputed datasets.** AUC (95% CI) are shown along with best cut off determined by Youden’s index along with respective sensitivity and specificities.

**Figure S34 - Receiver operating characteristics curves of support vector machine linear kernel trained with demographics, haematology and biochemistry and metabolome features to classify MASLD risk (cases defined by MRI-PDFF electronic health records) across five imputed datasets.** AUC (95% CI) are shown along with best cut off determined by Youden’s index along with respective sensitivity and specificities.

**Figure S35 - Receiver operating characteristics curves of logistic regression trained with demographics, haematology and biochemistry and metabolome features to classify MASLD risk (cases defined by MRI-PDFF electronic health records) across five imputed datasets.** AUC (95% CI) are shown along with best cut off determined by Youden’s index along with respective sensitivity and specificities.

**Figure S36 – Classification probabilities of machine learning models trained with selected demographic and haematology and biochemistry features.** Histogram shows probabilities for imputed data set one for descriptive purposes: (A) partial least square regression discriminant analysis, (B) random forest, (C) support vector machine linear kernel and (D) logistic regression against MRI-PDFF and diagnostic code MASLD case definition (left column of panel) and MRI-PDFF (right column of panel) are shown. Left histogram of each individual plot shows probabilities of healthy control and right show diseased individuals

**Figure S37 – Classification probabilities of machine learning models trained with selected demographic and haematology and biochemistry, and metabolome features.** Histogram shows probabilities for imputed data set one for descriptive purposes: (A) partial least square regression discriminant analysis, (B) random forest, (C) support vector machine linear kernel and (D) logistic regression against MRI-PDFF and diagnostic code MASLD case definition (left column of panel) and MRI-PDFF (right column of panel) are shown. Left histogram of each individual plot shows probabilities of healthy control and right show diseased individuals
